# Liquid Structure of Ionic Liquids with [NTf_2_]^−^ Anions, Derived from Neutron Scattering

**DOI:** 10.1021/acs.jpcb.3c08069

**Published:** 2024-03-23

**Authors:** Anne McGrogan, Jack Lafferty, Lauren O’Neill, Lucy Brown, J. Mark. Young, Peter Goodrich, Mark J. Muldoon, Leila Moura, Sarah Youngs, Terri-Louise Hughes, Sabrina Gärtner, Tristan G. A. Youngs, John D. Holbrey, Małgorzata Swadźba-Kwaśny

**Affiliations:** † QUILL Research Centre, Queen’s University Belfast, School of Chemistry and Chemical Engineering, David Keir Building, 39-123 Stranmillis Road, Belfast BT9 5AG, Belfast, U.K.; ‡ Rutherford Appleton Laboratory, Chilton, Didcot OX11 0QX, U.K.

## Abstract

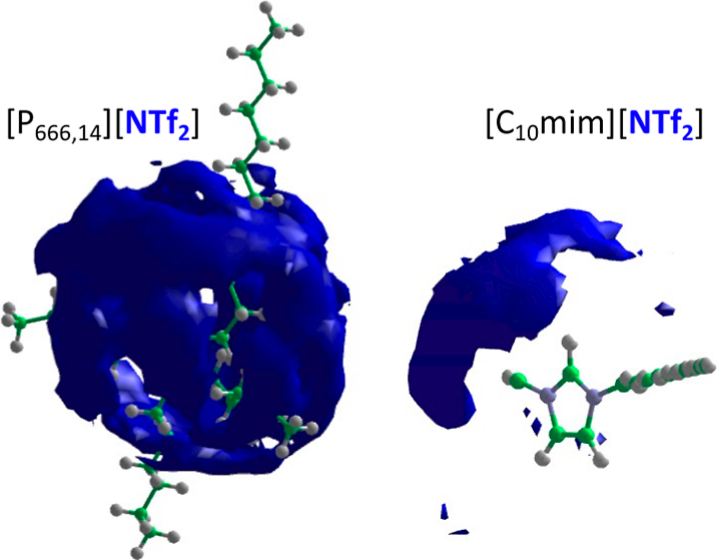

The liquid structure of three common ionic liquids (ILs) was investigated by neutron scattering for the first time. The ILs were based on the bis(trifluoromethanesulfonyl)imide anion, abbreviated in the literature as [NTf_2_]^−^ or [TFSI]^−^, and on the following cations: 1-ethyl-3-methylimidazolium, [C_2_mim]^+^; 1-decyl-3-methylimidazolium, [C_10_mim]^+^; and trihexyl(tetradecyl)phosphonium, [P_666,14_]^+^. Comparative analysis of the three ILs confirmed increased size of nonpolar nanodomains with increasing bulk of alkyl chains. It also sheds light on the cation–anion interactions, providing experimental insight into strength, directionality, and angle of hydrogen bonds between protons on the imidazolium ring, as well as H–C–P protons in [P_666,14_]^+^, to oxygen and nitrogen atoms in the [NTf_2_]^−^. The new Dissolve data analysis package enabled, for the first time, the analysis of neutron scattering data of ILs with long alkyl chains, in particular, of [P_666,14_][NTf_2_]. Results generated with Dissolve were validated by comparing outputs from three different models, starting from three different sets of cation charges, for each of the three ILs, which gave convergent outcomes. Finally, a modified method for the synthesis of perdeuterated [P_666,14_][NTf_2_] has been reported, with the aim of reporting a complete set of synthetic and data processing approaches, laying robust foundations that enable the study of the phosphonium ILs family by neutron scattering.

## Introduction

Ionic liquids (ILs) are salts that have melting points below 100 °C and often below ambient temperature. They are characterized by complex intermolecular interactions through Coulombic and van der Waals forces, as well as a rich network of hydrogen bonds. Elucidation of the liquid structure gives an important indication as to which of these interactions dominate in each IL, which in turn sheds light on the origin of their physicochemical properties, such as solvating power, density, and viscosity. It also gives insights into the microscopic heterogeneity of ILs (nanostructure): the segregation of the polar domains (the cation head and the anion) and nonpolar regions (the alkyl chains).^1,2^ The liquid structure of many IL families has been explored by X-ray^3−6^ and neutron scattering^7,8^ and molecular dynamics (MD).^9−13^

In the most common ILs, cations are based on quaternized nitrogen or phosphorus bases. The 1-alkyl-3-methylimidazolium cations, [C_*n*_mim]^+^, have served as workhorse IL cations for decades since their propensity to give room-temperature molten salts with chloroaluminate anions has been reported by Hussey^14^ and then extended to air- and water-stable ILs.^15,16^ Tetraalkylphosphonium ILs are of interest as they have a wide liquidus range and a relatively high thermal and electrochemical stability, making them attractive options for energy storage applications (batteries and supercapacitors)^17−19^ and in ammonia generation.^20^ They are also hydrophobic, which has sparked interest in their use in liquid–liquid separations, from metals to biomass.^21−28^

**Figure 1:**
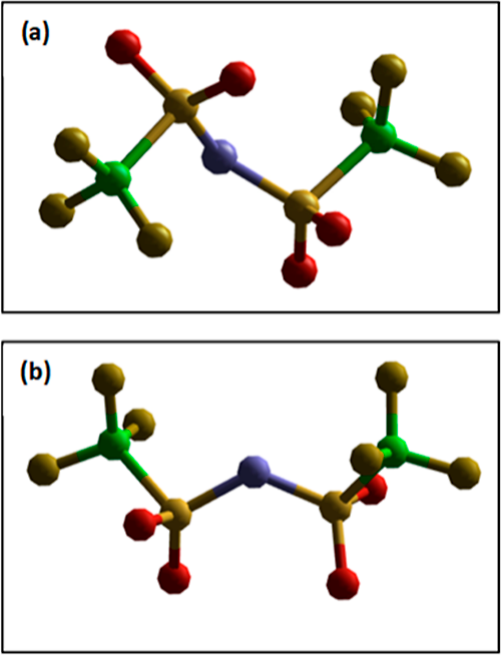
Models showing (a) the *trans* and (b) the *cis* configuration of the bis(trifluoromethanesulfonyl)imide anion, [NTf_2_]^−^.

The bis(trifluoromethanesulfonyl)imide anion, [NTf_2_]^−^, imparts a number of beneficial properties to ILs, including, but not limited to, a low melting point, relatively low viscosity, wide electrochemical window, hydrophobicity, and stability toward hydrolysis. On the molecular level, [NTf_2_]^−^ is characterized by diffuse charge distribution, very weak coordination ability (although it can coordinate to metals and accept hydrogen bonds via both N and O atoms), and the ability to adopt two conformations: *cis* and *trans* (Figure 1), all of which contribute to the low lattice energy of [NTf_2_]^−^ salts. This combination of properties has inspired a multitude of studies on the fundamental properties and applications of [C_*n*_mim][NTf_2_]^29−32^ and [P_666,14_][NTf_2_]^5,6,13,33−36^ ILs. Here, we study these ILs by neutron scattering because their liquid structure is vastly different from the solid. This was demonstrated by Deetlefs et al.^29^ in 2006 who showed that the liquid structure of [C_1_mim][NTf_2_], obtained from neutron scattering, had little correlation with its crystal structure.^37^ This was in contrast to the chloride and hexafluorophosphate salts, and the difference was attributed to the conformational flexibility of the [NTf_2_]^−^ anion, which can adopt both *cis* and *trans* conformers in the liquid state, in contrast to fixed conformers in the solid.^38^

**Figure 2:**
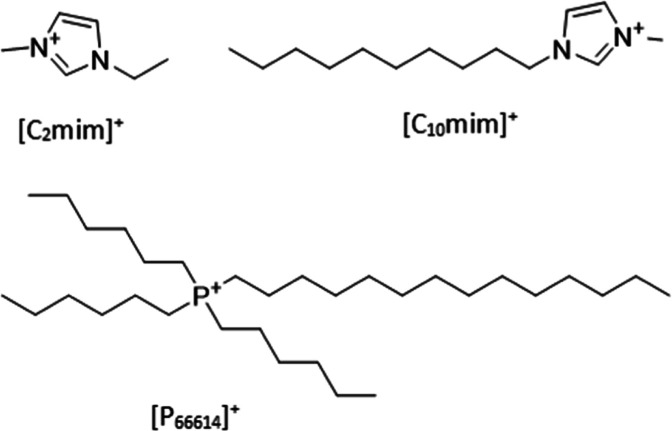
Structure of the three cations studied in this work: [C_2_mim]^+^, [C_10_mim]^+^, and [P_666,14_]^+^.

It is known that ILs are highly structured media, as far as the structure of liquids is concerned (that is, in short and medium range), which is driven by Coulombic interactions.^2,4,39−42^ The presence of long alkyl chains induces microsegregation into polar and nonpolar domains, which translates to differences in physicochemical properties.^9,43−45^ It can therefore be expected that an experimental neutron scattering study of the liquid structure of ILs with the same anion but three different cations: [C_2_mim][NTf_2_], [C_10_mim][NTf_2_], and [P_666,14_][NTf_2_] (Figure 2), will add fundamental understanding to structure–property relationships. Here, we report for the first time the structure of all three ILs by neutron scattering.

Over the past two decades, the use of the Empirical Potential Structure Refinement (EPSR) package, developed by Soper,^46^ to analyze neutron scattering data has been a valuable and indispensable tool. A new code, Dissolve, builds on the success of EPSR but employs a full classical force field and has the capability for million-atom simulations.^47^ More information on the development of the code and its capabilities is reported in a recent article introducing Dissolve.^47^ Dissolve was built to enable the analysis of larger and flexible structures more accurately. This paper aims to demonstrate the suitability of the Dissolve methodology for the analysis of long-chain ILs. At the time of writing, this was the first report of using Dissolve to study the liquid structure of ILs. To demonstrate the robustness of Dissolve analysis, three different sets of cation charges were used for the three ILs, and the models were shown to converge on the same outcome. This demonstrated the relative insensitivity of the simulation to these reasonable charge models.

Neutron scattering data for [C_2_mim][NTf_2_], [C_10_mim][NTf_2_], and [P_666,14_][NTf_2_] were recorded with H/D isotopic substitution. Along with demonstrating the suitability of the Dissolve methodology for the analysis of long-chain ILs, we report on a modified method for the synthesis of fully deuterated D_68_-[P_666,14_][NTf_2_]. We therefore demonstrate the overcoming of both modeling and synthetic barriers for the study of ILs with long alkyl chains, which we hope will open up a new strand of neutron scattering studies. Ultimately, the combination of Dissolve and appropriate deuteriation techniques is hoped to bridge the gap between simulations and experimental studies into the liquid structure.

Neutron and X-ray scattering experiments and MD studies have shown that the structure of ILs is dominated by strong cation–anion interactions through Coulombic forces as well as heterogeneity at the microscopic level: segregation of polar (*e.g*., the cation polar head and the anion) and nonpolar regions (alkyl chains).^3,9−12^ A comparative study was deemed interesting as modeling and X-ray^3,6,9,31,33,48,49^ scattering experiments indicate that long-chained ILs such as [C_10_mim][NTf_2_] and [P_666,14_][NTf_2_] will exhibit substantial nanosegregation compared to shorter-chain ILs such as [C_2_mim][NTf_2_]. Furthermore, protons on the imidazolium ring in [C_2_mim][NTf_2_] and [C_10_mim][NTf_2_], as well as H–C–P protons in [P_666,14_][NTf_2_], are expected to participate in hydrogen bonding, with oxygen and nitrogen atoms in the [NTf_2_]^−^ anion acting as hydrogen bond acceptors; these interactions can be quantified through a neutron scattering study.

## Experimental Section

### Materials and Methods

#### Synthesis of 1-Alkyl-3-methylimidazolium Bis(trifluorosulfonyl)imide ILs

[C_2_mim][NTf_2_] and [C_10_mim][NTf_2_] were synthesized according to standard procedures,^50^ details of which can be found in the Supporting Information. Fully deuterated [C_2_mim][NTf_2_] and [C_10_mim][NTf_2_] were synthesized by the Deuteration Facility at ISIS Neutron and Muon Source, STFC Rutherford Appleton Laboratory in Oxfordshire, UK, and used as received. Equimolar mixtures of protiated and deuterated components (H/D) were prepared on site at ISIS, prior to the neutron scattering experiments. Extensive experimental details for all synthetic steps as well as analytical data are given in the Supporting Information.

#### Synthesis of Trihexyl(tetradecyl)phosphonium Bis(trifluorosulfonyl)imide ILs

[P_666,14_][NTf_2_] was synthesized according to a previously reported procedure;^51^ see Supporting Information.

D_68_-[P_666,14_]Cl was synthesized according to a modified procedure by Atkin et al.^52^ Commercially available D_29_-1-tetradecanol was converted to D_29_-1-tetradecyl chloride using thionyl chloride. Commercially available trihexylphosphine was deuterated via Pd/C-catalyzed H/D exchange reaction in D_2_O, followed by reduction using phenylsilane. D_68_-tri(hexyl)tetradecylphosphonium chloride was then synthesized through alkylation. Finally, [P_666,14_][NTf_2_] was synthesized by routine ion exchange with Li[NTf_2_]. The detailed experimental procedure is reported here; NMR spectra and mass spectrometry data are provided in the Supporting Information.

#### Synthesis of D_29_-1-Tetradecyl Chloride

Thionyl chloride (13.93 g, 0.117 mol) was added to a two-necked round-bottomed flask equipped with a PTFE-coated magnetic stirrer bar, condenser, and pressure-equalizing dropping funnel, which were both fitted with calcium chloride guard tubes. D_29_-tetradecanol (9.50 g, 0.0390 mol) was then added to the pressure-equalizing dropping funnel and added slowly to the thionyl chloride with stirring. As the reaction progressed, both heat and SO_2_ evolved. When all the alcohol was added, the mixture was heated at reflux for 3 h. The excess of thionyl chloride was then separated from the product by distillation (78–80 °C), with the crude 1-tetradecyl chloride requiring 98 °C and 0.5 mbar. This was then washed with D_2_O, 10% sodium carbonate solution, and twice with D_2_O and then dried with anhydrous calcium chloride and distilled again (8.31 g, 81% yield, deuteration level 99% calculated by quantitative ^1^H NMR).

#### Synthesis of D_39_-Trihexylphosphine Oxide

The reaction was carried out in a 100 mL Parr high pressure reactor fitted with a mechanical stirrer. A mixture of trihexylphosphine (3.48 g, 0.0121 mol), 10 wt % Pt/C catalyst (0.75 g), and 10 wt % Pd/C catalyst (0.75 g) in D_2_O (65 mL) were added to the 100 mL Parr reactor body (made of Hastelloy c276), followed by N_2_ bubbling for 2 min and then H_2_ bubbling for 2 min at room temperature. After the reactor was sealed, the reactor was pressurized with nitrogen (40–50 bar). The reactor was then heated to 220 °C using the heating mantle and stirred at 600 rpm for 24 h. After cooling, dichloromethane was added to the reaction mixture, filtered through Celite, and washed with dichloromethane, and then the aqueous phase was extracted with dichloromethane (3 × 50 mL). The combined extracts were dried over MgSO_4_ and then evaporated to give deuterated D_39_-trihexylphosphine oxide as a white solid (1.72 g, 41% yield, deuteration level of 96%, calculated by quantitative ^1^H NMR). This synthesis was repeated, and batches were combined.

#### Synthesis of D_39_-Trihexylphosphine

In a 25 mL round-bottom flask fitted with a condenser, D_39_-trihexylphosphine oxide (4.49 g, 0.0148 mmol) was dissolved in phenylsilane (6 mL) under an atmosphere of argon and then heated to 100 °C overnight with stirring. The reaction was monitored by ^31^P NMR, by taking a sample from the reaction mixture and dissolving degassed CDCl_3_. The disappearance of the starting material peak at 50.8 ppm and the appearance of the D_39_-trihexylphosphine peak at −33.65 ppm confirmed that the reaction was complete. Phenylsilane was removed under reduced pressure to give a pale yellow residue, which is used in the next step without further purification.

#### Synthesis of D_68_-Trihexyltetradecylphosphonium Chloride

D_39_-trihexylphosphine (4.50 g, 0.0127 mol) and D_29_-tetradecyl chloride (4.32 g, 0.0165 mol) were mixed together and heated to 143 °C in a sealed tube in an argon-filled glovebox for 24 h. A white solid was present in the tube, along with the IL. The liquid was decanted, and ^31^P NMR of the liquid in CDCl_3_ showed that the ^31^P NMR peak of D_39_-trihexylphosphine at −33.65 ppm had disappeared to give a peak at 32 ppm, indicating the formation of D_68_-[P_66614_]Cl. The white solid was not fully soluble in common NMR solvents, and this was attributed to the formation of 1,3-diphenyl-disiloxane (PhH_2_Si–O–SiH_2_Ph) that would have formed in the previous step. This was further verified by X-ray fluorescence (XRF) analysis, which confirmed the presence of silicon, reporting a value of 82 000 ppm. This was not a high precision calibration but an internal calibration. XRF analysis of the IL did not detect any silicon. The solid was extracted with chloroform and filtered. Chloroform and excess alkylating agent were removed under vacuum.

#### Synthesis of D_68_-Trihexyltetradecylphosphonium Bis(trifluoromethylsulfonyl)imide

D_68_-[P_666,14_]Cl (0.010 mol equiv) was dissolved in hexane and Li[NTf_2_] (0.012 mol equiv) was dissolved in D_2_O, combined in a round-bottomed flask, and left to stir for 3 h at room temperature, 600 rpm. The organic layer was separated and washed multiple times with D_2_O. Hexane was removed, and the IL was dried under high vacuum (12 h, 70 °C, 10^–2^ mbar) to give a colorless liquid (5.67 g, 94% yield, deuteration level 97%). XRF analysis confirmed that the chloride content was below the detectable limit.

#### Neutron Scattering Experiments

All measured ILs were dried (<200 ppm of water by Karl Fischer measurements), transferred to an argon-filled glovebox, placed in ampules, and flame-sealed glovebox and placed in ampules. The ampules were flame-sealed, removed from the glovebox and shipped to the ISIS at Rutherford Appleton Laboratory, Oxfordshire, UK. There, the samples were transported into a glovebox and transferred to oven-dried 1 mm “zero scattering” Ti_0.68_Zr_0.32_ flat plate cells, which were sealed before their removal from the glovebox into open air.

**Figure 3:**
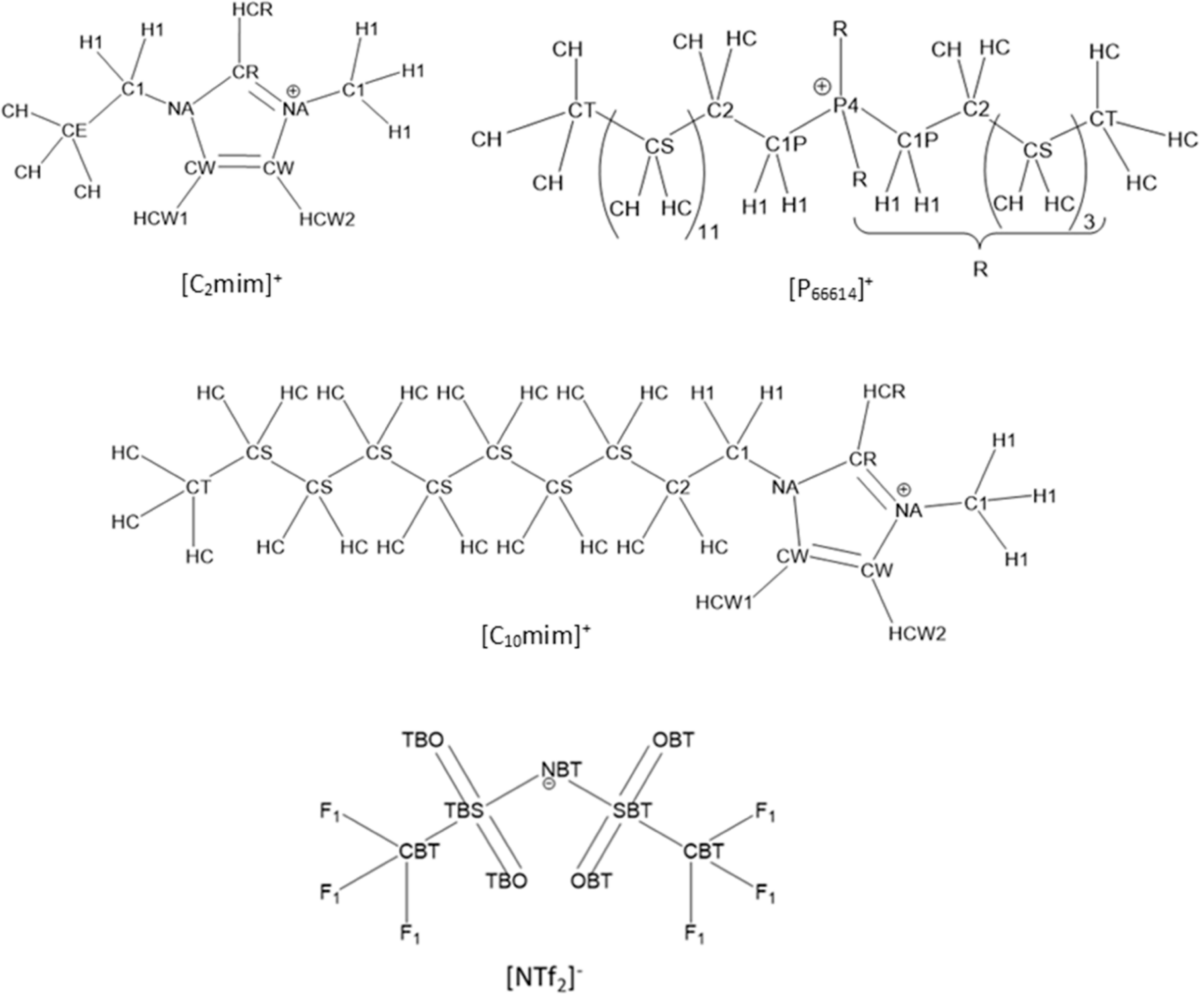
Molecular structures and atom types for the [C_2_mim]^+^, [C_10_mim]^+^, and [P_666,14_]^+^ cations and [NTf_2_]^−^ anion.

Neutron scattering data for the three ILs were recorded at 25 °C using the Near and InterMediate Range Order Diffractometer (NIMROD) and the Small Angle Neutron Diffractometer for Amorphous and Liquid Samples (SANDALS) instruments at ISIS. Data reduction encompassing removal of container and instrument backgrounds, corrections for multiple scattering and attenuation, and the removal of inelasticity effects was performed with the Gudrun software.^53^ The neutron diffraction data were fitted and analyzed using Dissolve software across the entire Q-range. Dissolve performs empirical potential structure refinement using a methodology similar to that employed in the EPSR25 code by Soper.^46^ Molecular structures and atom types for the [C_2_mim]^+^, [C_10_mim]^+^, and [P_666,14_]^+^ cations and [NTf_2_]^−^ anion are shown in Figure 3.

Simulation box sizes contained 500 ion pairs for [C_2_mim][NTf_2_] and [C_10_mim][NTf_2_] and 250 ion pairs for [P_666,14_][NTf_2_]. The force-field parameters were taken from the Canongia Lopes & Padua (CL&P) force field.^54−56^ The three sets of cation charges are given in Tables S3–S5. Set 1 was from the CL&P force field. Set 2 was electrostatic potential (ESP) charges calculated with the NWChem software (v7.0.2). Geometry optimizations were performed on all ions at increasing basis sets up to HF/6-31+G(d), at which point the ESP charges were calculated using the standard module defaults. Resulting charges were averaged across symmetry-related and/or chemically equivalent sites on the molecules, and significant figures truncated to three in order to provide manageable charges for the simulation, always ensuring that the total charge remained at ±1. Set 3 charges were generated from the LigParGen service offered by the Jorgensen group.^57−59^

## Results and Discussion

This article covers three aspects of the study of [NTf_2_]^−^ ILs by neutron scattering. First, a short discussion of the synthesis of perdeuterated [P_666,14_][NTf_2_] is provided. Second, the structure of the three liquids is discussed, elucidated by data-driven modeling of neutron scattering data using the Dissolve package. Finally, considering that this is the first paper reporting the use of Dissolve for ILs, there is a discussion about the robustness of the method, which is probed by comparative simulations using three different sets of potentials.

### Synthesis of D_68_-[P_666,14_][NTf_2_]

**Scheme 1:**
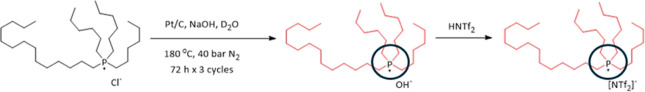
Direct Deuteration of the [P_666,14_]^+^ Cation of [P_666,14_]Cl, Yielding a Hydroxide IL, Followed by Acid/Base Neutralization with HNTf_2_ Black alkyl chains show the protonated chains and the red alkyl chains depict the deuterated alkyl chains. The black circles indicate the protons that were successfully exchanged.

Three approaches to the synthesis of perdeuterated [P_666,14_][NTf_2_] were attempted (see Supporting Information for full experimental methods). Method 1 was the direct deuteration of the [P_666,14_]^+^ cation of [P_666,14_]Cl, which yielded a hydroxide IL, followed by acid/base neutralization with HNTf_2_ (Scheme 1).

The first step was carried out by subjecting [P_666,14_]Cl to multiple cycles of hydrothermal H/D exchange in D_2_O, under basic conditions, catalyzed with Pt/C, which yielded the [P_666,14_][OH] solution in D_2_O. This solution, after filtration, was neutralized with HNTf_2_ to generate [P_666,14_][NTf_2_]. The product gave one strong NMR signal at δ_31P_ = 32 ppm (Figure S12), but despite multiple cycles, only the eight protons in the α position with respect to the phosphorus atom (H–C–P) were deuterated, as confirmed by ^1^H NMR spectroscopy (Figure S11) and mass spectrometry (Figure S14). An attempt to remove D_2_O under reduced pressure prior to the neutralization step has resulted in decomposition of the cation with the formation of an ylide, as indicated by ^31^P NMR spectroscopy (see Figure S36).

**Scheme 2:**
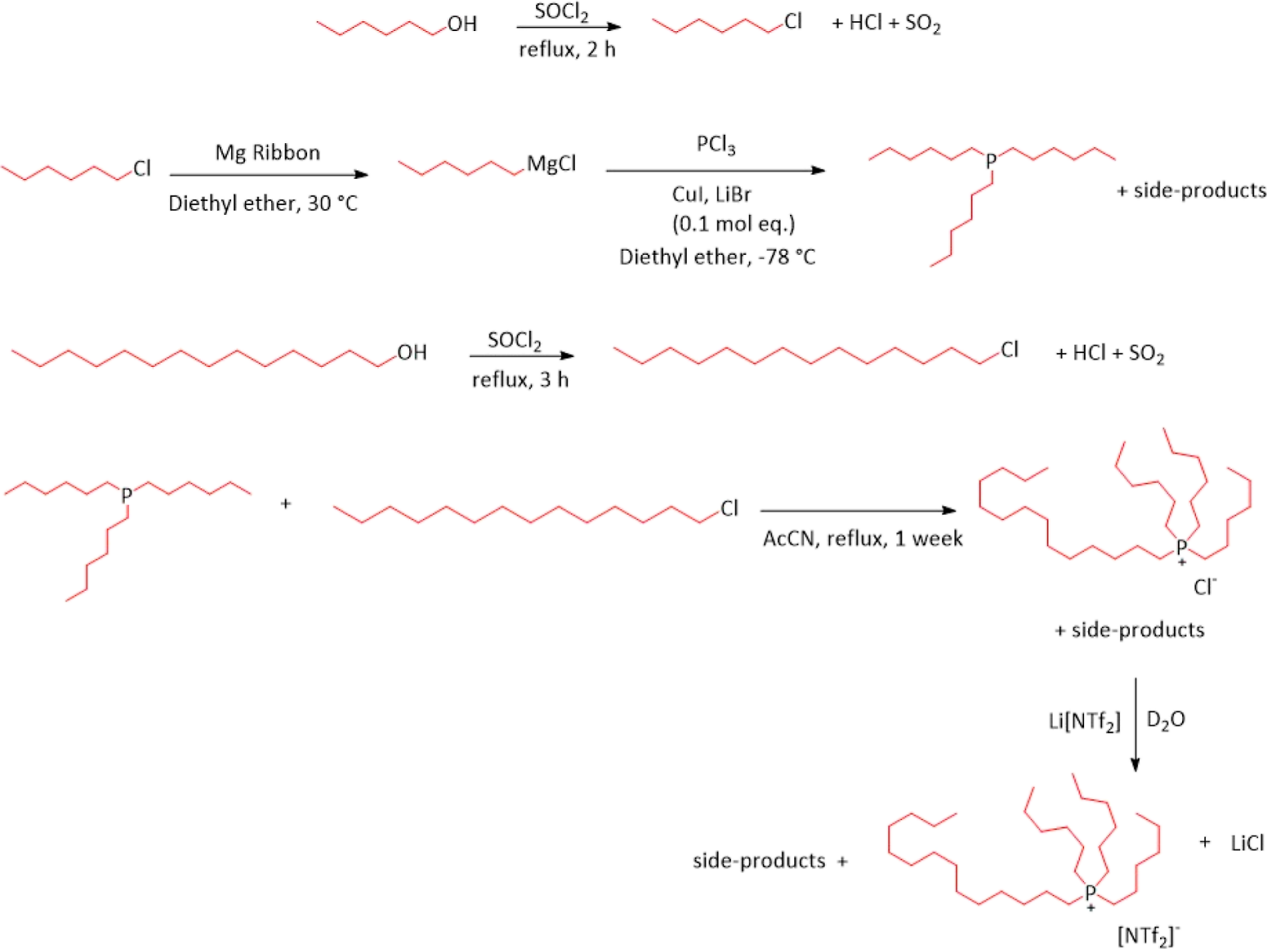
Synthesis of Perdeuterated Trihexylphosphine, D_39_-P_666_, via Grignard Reaction, Followed by Alkylation with D_29_-1-Chlorotetradecane to Yield D_68_-[P_666,14_]Cl This was then subjected to ion exchange with Li[NTf_2_] to generate D_68_-[P_666,14_][NTf_2_]. Red alkyl chains depict the deuterated alkyl chains.

Method 2 involved the synthesis of D_13_-1-chlorohexane and D_29_-1-chlorotetradecane by chlorination of the corresponding alcohols with thionyl chloride. Then, the synthesis of perdeuterated trihexylphosphine, D_39_-P_666_, was done from D_13_-1-chlorohexane via Grignard reaction, followed by alkylation with D_29_-1-chlorotetradecane to yield D_68_-[P_666,14_]Cl. This would then be subjected to ion exchange with Li[NTf_2_] to generate D_68_-[P_666,14_][NTf_2_] (Scheme 2).

The method to synthesize the perdeuterated trihexylphosphine has been adapted from a synthetic procedure for perdeuterated tri-*tert*-butylphosphine, reported by some of us prior to this publication.^60^ Unfortunately, despite repeated attempts and meticulous work under an inert atmosphere, the procedure yielded a mixture of multiple species, as indicated by ^31^P NMR spectroscopy (Figure S19). In addition to several signals attributable to phosphines, products of oxidation have been found, including trihexylphosphine oxide, which eventually led to the abandonment of this method.

**Scheme 3:**
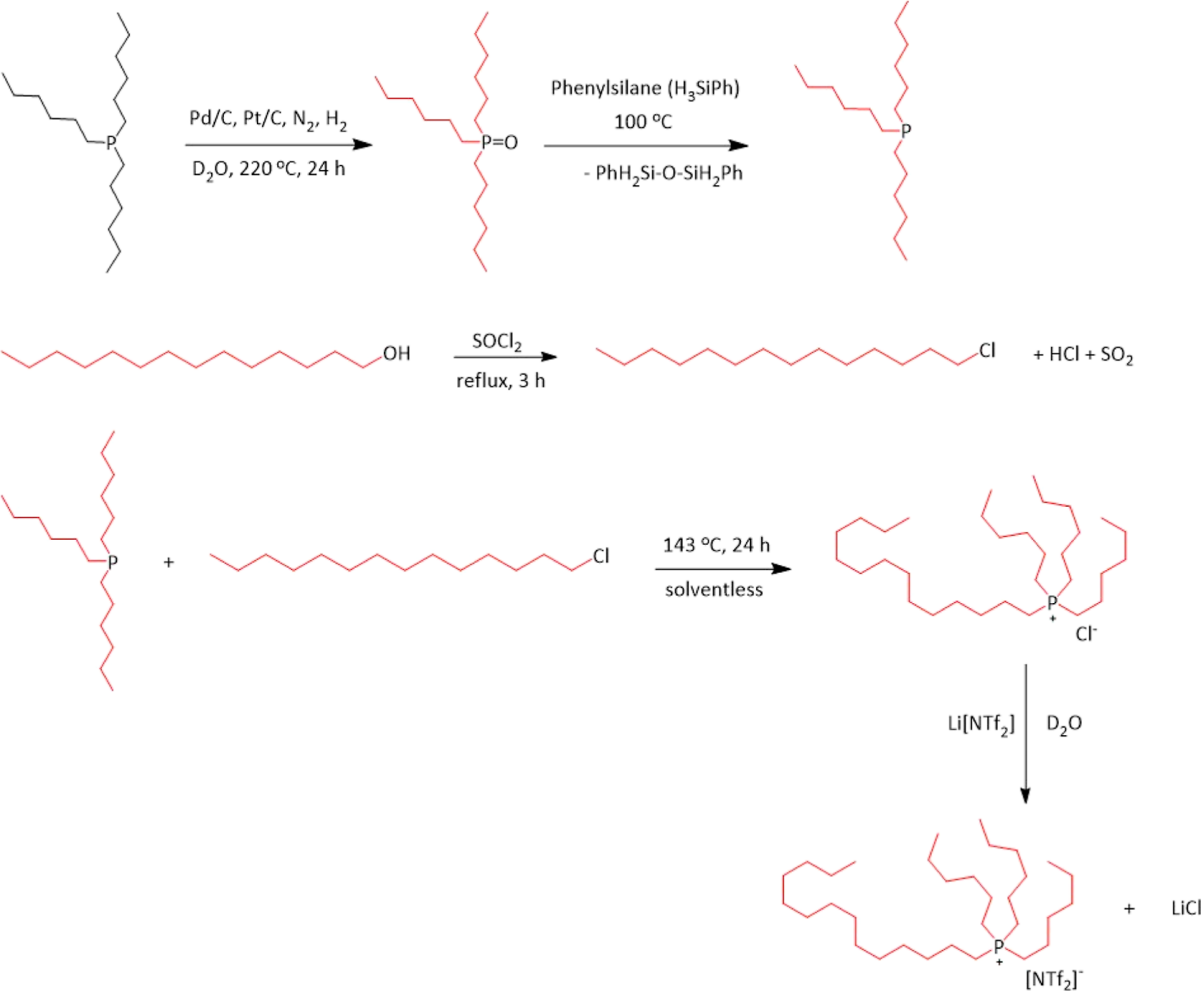
Synthesis of D_68_-Trihexyltetradecylphosphonium Bis(trifluoromethylsulfonyl)amide Black alkyl chains show the protonated chains and the red alkyl chains depict the deuterated alkyl chains.

Method 3 (Scheme 3, see Supporting Information for full details) relied on the catalytic deuteriation of trihexylphosphine in D_2_O, with perdeuterated trihexylphosphine oxide as the product. The phosphine oxide was subsequently reduced and then alkylated with D_29_-1-chlorotetradecane, followed by anion exchange with Li[NTf_2_], as shown in Scheme 3. The procedure was based on the method reported by Atkin et al.,^52^ with several key modifications. First, D_29_-1-chlorotetradecane was synthesized by the chlorination of the corresponding alcohol with thionyl chloride, rather than using *n*-chlorosuccinimide and triphenylphosphine, eliminating the need for purification using column chromatography. Second, whereas Atkin et al. used column chromatography to purify D_68_-[P_666,14_]Cl, here it has been possible to convert the crude D_68_-[P_666,14_]Cl directly into pure D_68_-[P_666,14_][NTf_2_], leaving all side products in the aqueous phase.

**Figure 4:**
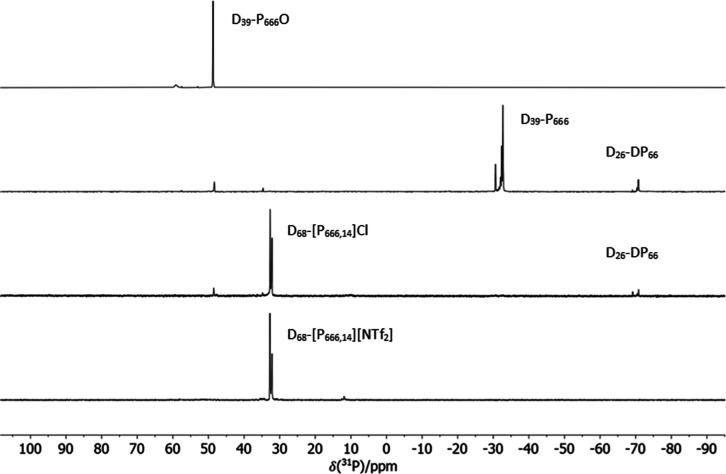
^31^P NMR spectra of the compounds synthesized at consecutive steps of the synthesis of D_68_-[P_666,14_][NTf_2_], with assignments of the products and impurities.

Deuteration of P_666_ was carried out using a combination of Pt/C and Pd/C catalysts in D_2_O, with the reaction carried out at 220 °C for 24 h in a pressurized reactor vessel. It has previously been reported that this catalyst system is effective for the deuteration of other substrates.^61^ The product was a white solid that gave one strong NMR signal of δ_31P_ = 48.8 ppm (Figure 4), with a deuterium content of 96%, as calculated by quantitative ^1^H NMR spectroscopy. This was further confirmed by mass spectrometry (Figure S24), which gave an average *m*/*z* value of 341, which is consistent with deuteration and oxidation to D_39_-P_666_O. D_39_-P_666_O was then reduced using phenylsilane, and the reaction was monitored by ^31^P NMR spectroscopy in degassed CDCl_3_, following the disappearance of the starting material peak at δ_31P_ = 48.8 ppm and the appearance of the D_39_-P_666_ peak at δ_31P_ = −32.6 ppm (Figure 4). A side-product formation (δ_31P_ = −70.9 ppm), accounting for *ca*. 11% of the product mixture, has been observed, in agreement with the report by Atkin et al.^52^ This was attributed to dihexylphosphine, in agreement with the literature.^62^ Following the alkylation step, D_68_-[P_666,14_]Cl contained impurities associated with D_39_-P_666_O and D_26_-DP_66_; both were removed with multiple aqueous washes upon the ion exchange step (Figure 4).

### EPSR Modelling Using Dissolve and Fit to Experimental Data

**Figure 5:**
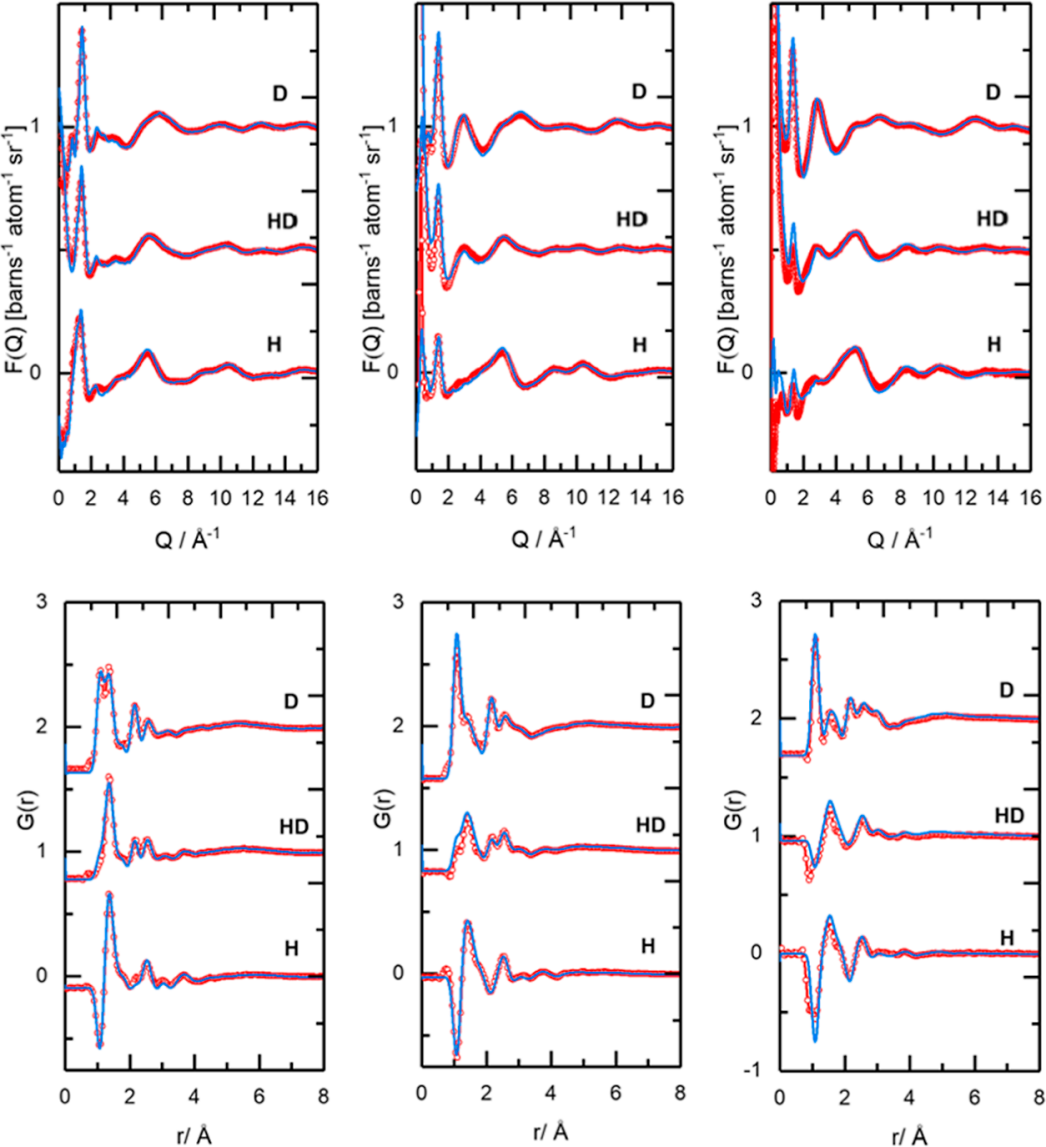
Total structure factors *F*(*Q*) (top) and the corresponding Fourier transforms to real space *G*(*r*) RDFs (bottom) showing experimental data (red symbols) and Dissolve modeled (blue solid line) data for left: [C_2_mim][NTf_2_], middle: [C_10_mim][NTf_2_], and right: [P_666,14_][NTf_2_].

Total neutron scattering data for [C_2_mim][NTf_2_], [C_10_mim][NTf_2_], and [P_666,14_][NTf_2_], each measured at three levels of isotopic substitutions (H, D, H/D), were reduced using the GUDRUN^53^ package and modeled using Dissolve.^47^ The experimental sample densities and scattering levels were consistent with the correct isotopic compositions of the samples. Comparisons of experimental and simulated total structure factors, *F*(*R*), and the corresponding Fourier transforms to real space, *G*(*r*), for the three ILs at three substitution levels, measured at ambient temperature, are shown in Figure 5. Apart from the region at *Q* ≤ 1 Å^–1^, which is most susceptible to inconsistencies due to inelastic scattering contributions from hydrogen in the data, the fitted data align well with experiment.

### Center-of-Mass Radial Distribution Functions

**Figure 6:**
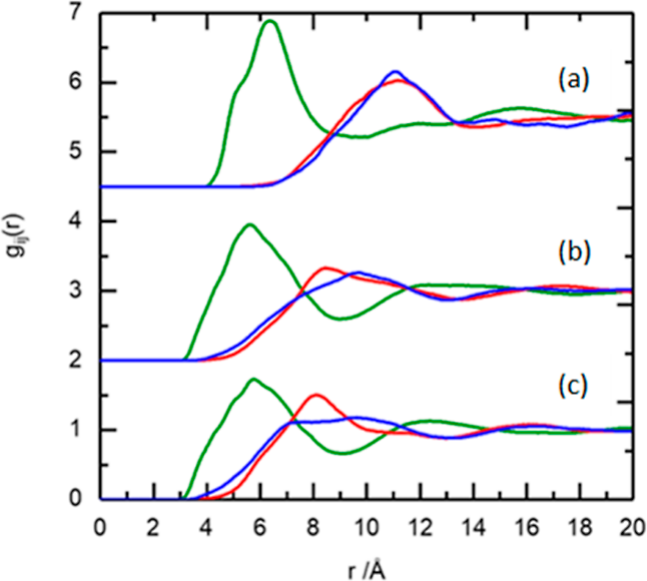
Comparison of the RDFs for the cation–anion distribution (green line), the cation–cation distribution (blue line), and the anion–anion distribution (red line) for (a) [P_666,14_][NTf_2_], (b) [C_10_mim][NTf_2_], and (c) [C_2_mim][NTf_2_].

The most important information about the IL structure—the ordering of cations and anions with respect to one another—can be extracted from the radial distribution functions (RDFs), describing the distribution of atoms and species around a central point. In ILs, these tend to be the nominal centers of charge. Following the convention, the central points selected in this work were as follows: the phosphorus of [P_666,14_]^+^, the center of mass taken from the midpoint of the two nitrogen atoms of the imidazolium ring in [C_2_mim]^+^ and [C_10_mim]^+^, and the nitrogen of [NTf_2_]^−^. RDFs describing the cation–anion distributions for the three [NTf_2_]^−^ ILs are shown in Figure 6.

The cation–anion contact distance (green curves in Figure 6) is centered at ∼6 Å for all three ILs, similarly to the distances reported for other ILs^7,29,49,63–65^ and irrespective of the size difference between [C_2_mim]^+^, [C_10_mim]^+^, and [P_666,14_]^+^. Computational studies have been published on all three ILs: [P_666,14_][NTf_2_] was modeled by Parker et al.,^13^ [C_2_mim][NTf_2_] was studied by the groups of Fujii (MD)^30^ and Boero (DFT),^66^ and [C_10_mim][NTf_2_] was studied by Lopes et al.^31^ In all cases, the models were broadly similar to the neutron scattering results, but computational methods suggested the presence of a double peak for the first-shell correlation of the cation–anion: around 5 and 6 Å, attributed to *cis* and *trans* [NTf_2_]^−^ conformers. In neutron scattering data, these features were significantly less resolved: in [P_666,14_][NTf_2_], there was a slight shoulder at ∼5 Å, and a single, albeit broader, peak was recorded for [C_2_mim][NTf_2_] and [C_10_mim][NTf_2_]. Finally, several authors describe a low-intensity peak at 3.5 Å in calculated partial RDFs (pRDFs) for [C_2_mim][NTf_2_] and [C_10_mim][NTf_2_], which manifests itself in the neutron scattering data in the form of a broadening of the main feature at 6 Å (green curves in Figure 6b,c), which is absent from the analogous [P_666,14_][NTf_2_] curve (green curves in Figure 6a). This broad peak is from the shortest contact of anion to center of mass of the imidazolium ring cation where anion distribution is located above/below the ring. This distribution is due to the large size and charge delocalization of the [NTf_2_]^−^ anion, which has the effect of reducing the hydrogen bonding accepting ability of the anion, and thus the interaction with the ring hydrogens reduces, in contrast to smaller anions like chloride.^1^ This first cation–anion contact distance centered at ∼6 Å, which presents as a broad peak in the RDF, has also been observed in neutron scattering studies of several ILs: [C_1_mim][NTf_2_]^29^ and [C_4_mim][NTf_2_]^65^ and in a series of [C_*n*_mim][PF_6_] ILs, where *n* = 4, 6, and 8.^49^ The latter publication shows retention of the cation–anion first shell with changing cation alkyl chain length.

The cation–anion coordination numbers (CNs), calculated from the integration of the cation–anion RDFs up to the first minimum at 9 Å (green curves in Figure 6), were found to increase with decreasing cation size. In [P_666,14_][NTf_2_], there were three anions in the first shell of each cation, in agreement with MD studies by Liu and co-workers.^5^ CNs increased to 5 for [C_10_mim][NTf_2_] and further to 7 for [C_2_mim][NTf_2_], which is again comparable with the literature data.^66^

The cation–cation distance and CNs (P...P distribution), as well as anion–anion distance and CNs (N...N distribution), are nearly identical for [P_666,14_][NTf_2_], with maxima at ∼11 Å and CNs of 9 (to a distance of 14 Å). It is evident from blue and red curves in Figure 6a and remains in agreement with MD studies by Liu and co-workers.^5^ In [C_10_mim][NTf_2_] and [C_2_mim][NTf_2_], the peaks describing the anion–anion distances (red curves in Figure 6b,c) have maxima at much shorter distance (∼8.5 and 8.0 Å, respectively) but are much broader than the corresponding feature in [P_666,14_][NTf_2_]. In consequence, when integrated, they give CNs of 25 and 17, respectively. The peaks corresponding to cation–cation interactions for [C_10_mim][NTf_2_] and [C_2_mim][NTf_2_] (blue curves in Figure 6b,c) also have maxima at a shorter distance of ∼9 Å but again are much broader then the corresponding feature in [P_666,14_][NTf_2_].

The conformational flexibility and low basicity of [NTf_2_]^−^^1^, combined with the rotational freedom of the relatively small [C_2_mim]^+^ cation, resulted in very little long-range structure, apart from that imposed by the ordering of alternating charges. In particular, the nearly featureless blue curve in Figure 6c indicates a close to random orientation of [C_2_mim]^+^ cations. This is in agreement with a number of computational, Raman, IR, and UV–vis spectroscopic studies, which point to a large number of cation/anion orientations existing within a very small energy difference (<0.5 kcal/mol).^67,68^ In [C_10_mim][NTf_2_], the cation–anion distance is the same as in [C_2_mim][NTf_2_], despite the larger cation size. This suggests that the anion is positioned around the ring, and the long alkyl chain protrudes away from the charged region (does not contribute to cation–anion separation). While ring–anion interactions are analogous to [C_2_mim][NTf_2_], the decyl chain partially restricts the rotational freedom of the cation, enforcing more cationic ordering (blue curve in Figure 6b). The structure of in [P_666,14_][NTf_2_] can be envisaged as phosphonium point charges, arranged every 11 Å in all directions (CN 9), with alkyl chains that protrude from these cationic centers and overlap, attracted by van der Waals forces. Anions sit in holes between the alkyl chains, at 6 Å from the nearest cation (green curve in Figure 6a), the distance resulting from the interplay between Coulombic attraction and steric hindrance. For the weakly coordinating [NTf_2_]^−^ anion, long alkyl chains decrease the electrostatic attraction, thus increasing the attraction between the ion pairs. Ion pairing in phosphonium ILs, studied by pulse field gradient NMR spectroscopy, has been shown to increase with increasing alkyl chain length, which points to the high degree of ion pairing in [P_666,14_][NTf_2_].^69^ This, in turn, explains the existence of identical, well-pronounced cation–cation and anion–anion correlations as the direct consequence of ion pairing.

### Aggregate Analysis

**Figure 7:**
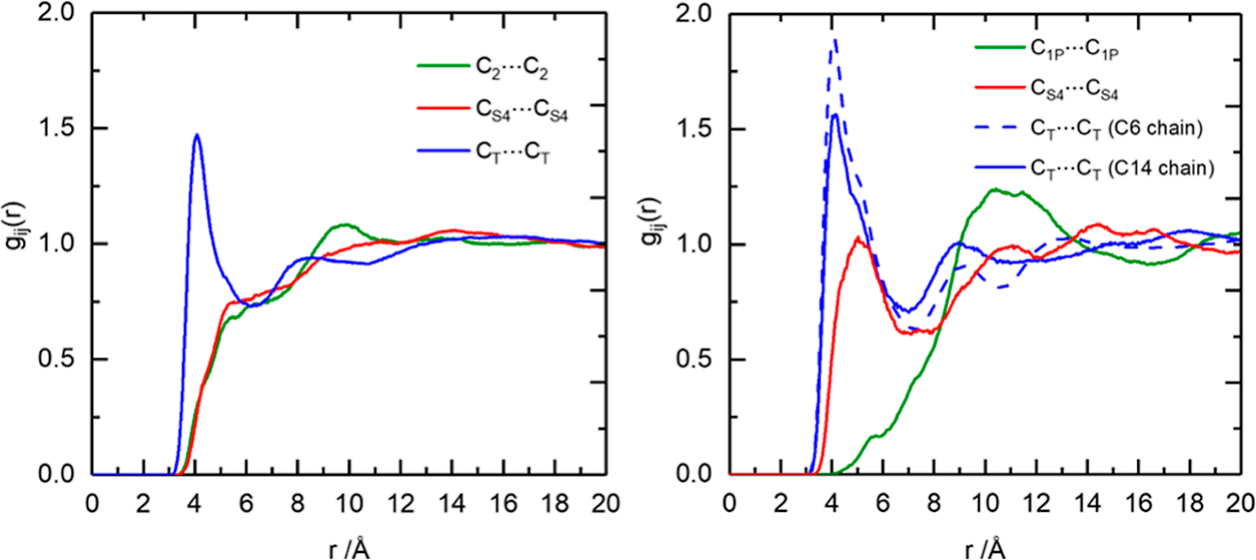
RDFs of carbon atoms along the alkyl chain in left: [C_10_mim][NTf_2_]—C_2_ (green), C_S4_ (red), and C_T_ (blue)—and right: [P_666,14_][NTf_2_]—C_1P_ (green), C_S4_ (red), and C_T_—of the C6 chain (blue-dashed) and C_T_ of the C14 chain (blue-solid).

To investigate the aggregation of the alkyl chains in [C_10_mim][NTf_2_] and [P_666,14_][NTf_2_], a comparison of the pRDFs for carbon atoms in the beginning (C_2_/C_1P_), middle (C_S4_), and terminal (C_T_) parts of the alkyl chain was plotted (Figure 7). For codes of atom types, refer to Figure 3.

The highest and the most intense peaks occurred for the terminal carbons (C_T_), demonstrating a strong spatial correlation between the nonpolar parts of hydrocarbon chains. All three C_T_ first shell peaks have maxima at a very short distance of 4 Å, CN = 2 for C_T_ in [C_10_mim][NTf_2_] and CN = 1 for both C_T_(C6) and C_T_(C14) of [P_666,14_][NTf_2_] (up to a distance of 7 Å). There is also evidence for a longer-range order, with pronounced second shell correlation peaks at 8–9 Å, which maps to typical cation–cation separation distance and shows evidence of longer length scale oscillation in the structure. These findings correspond to a MD simulation study on [C_10_mim]^+^ ILs with amino acid-derived anions.^70^ On the other hand, carbons adjacent to charged centers showed features at 10 Å for C_2_ in [C_10_mim][NTf_2_] and 11 Å for C_1P_ in [P_666,14_][NTf_2_], in both cases perfectly aligned with the corresponding cation–cation correlation (Figure 6).

It is known from the literature that [C_10_mim]Cl and its many hydrates have very ordered structures, with crystal packing containing double rows of charged imidazolium rings and chloride anions and nonpolar domains of overlapping alkyl chains (close contacts for both C_T_...C_T_ and C_4S_...C_4S_).^71^ In contrast, lack of order in the polar domain of [C_10_mim][NTf_2_] (see discussion under Figure 6) appears to result in alkyl chains protruding in different directions and therefore no ordering in C_4S_...C_4S_. Only the ends of decyl chains appear to assemble into nonpolar domains due to van der Waals forces, as shown by close C_T_...C_T_ contacts (4 Å, CN = 2, Figure 7 left). In contrast, the RDF middle-of-the-chain carbon of the C14 chain of [P_666,14_][NTf_2_] does show a peak at around 5 Å. This is aligned with the image proposed in the discussion under Figure 6, in which alkyl chains protrude from phosphonium point charges, arranged every 11 Å, resulting in the necessary overlap of these alkyl chains. Unsurprisingly, this suggests that the size of nonpolar domains in [P_666,14_][NTf_2_] is much larger than that in [C_10_mim][NTf_2_], not only due to increased volume of the alkyl chains but also due to their much better overlap.

### Hydrogen Bonding Analysis

**Figure 8:**
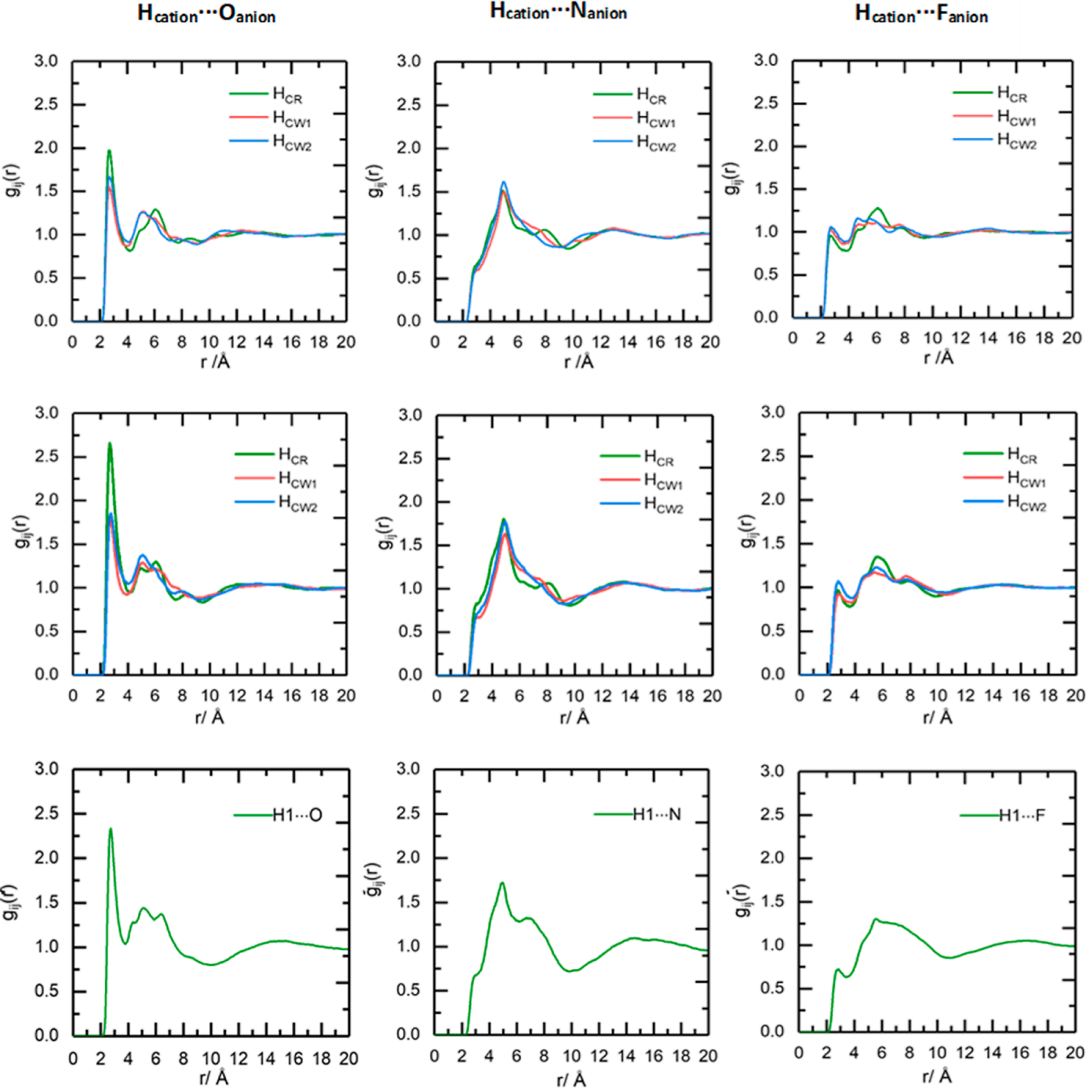
RDFs for top: [C_2_mim][NTf_2_], middle: [C_10_mim][NTf_2_], and bottom: [P_666,14_][NTf_2_] for interactions between the cation ring hydrogens/H_1_ and oxygen (left), nitrogen (middle), and fluorine atoms (right) of the anion. H_CW1_ refers to H_CW_ beside the alkyl chain and H_CW2_ refers to H_CW_ beside the methyl group.

To understand atom-specific interactions in the cation–anion association for the three ILs, which can inform about their chemistry and solvating properties, atom-specific pRDFs were derived. Correlations between the cation ring hydrogens (H_CR_ and H_CW_) of [C_*n*_mim]^+^, or the H_1_ protons of [P_666,14_]^+^, and the O, F, and N atoms of the [NTf_2_]^−^ anion are shown in Figure 8.

At first glance, the distributions are similar for each interaction, demonstrating the presence of strong hydrogen bonds. The H_cation_...O_anion_ interactions feature first-shell close contacts at ∼2.7 Å, with weaker and broader peaks at ∼5.5 Å. The H_cation_...N_anion_ interactions feature a slight shoulder at ∼2.7 Å, which is much less pronounced compared to the H_cation_...O_anion_ interaction. The main peak for H_cation_...N_anion_ is centered around 5 Å, which is the distance associated with the anion interacting via its oxygens. This corresponds to reports by Boero et al. and Liu and co-workers.^5,66^ In summary, the acidic hydrogens interact with the anion mainly through the O atoms and much less through the N atoms, and there is virtually no interaction via the F atoms in the first coordination sphere. The imidazolium cation and the [NTf_2_]^−^ anion preferentially interact through the H_CR_ atom in [C_*n*_mim]^+^, which is more acidic than H_CW_ (again, this is consistent with MD studies).^30,66^ CNs for H_cation_...O_anion_ are 0.4–0.5 for the [C_*n*_mim]^+^ ILs and only 0.2 for the H_1_ protons in [P_666,14_]^+^, which is smaller as interaction of each [NTf_2_]^−^ anion is averaged across eight H_1_ hydrogens. The relative distances and CNs of the key correlations for the three ILs are shown in Tables S1 and S2.

**Figure 9:**
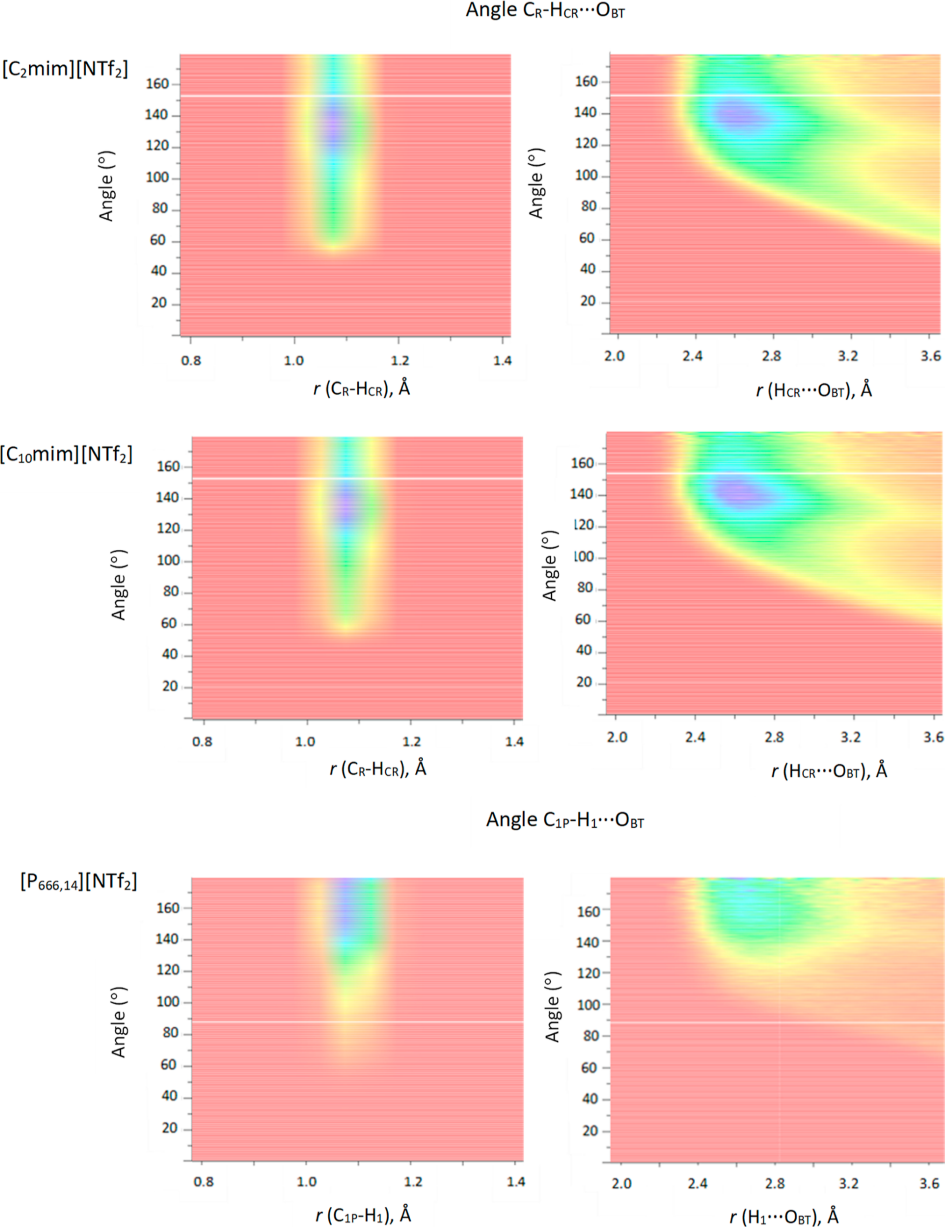
Distance and angle analysis of hydrogen bonds between atom types C_R_–H_CR_...O_BT_ for [C_2_mim][NTf_2_] and [C_10_mim][NTf_2_] and C_1P_–H_1_...O_BT_ for [P_666,14_][NTf_2_].

**Figure 10:**
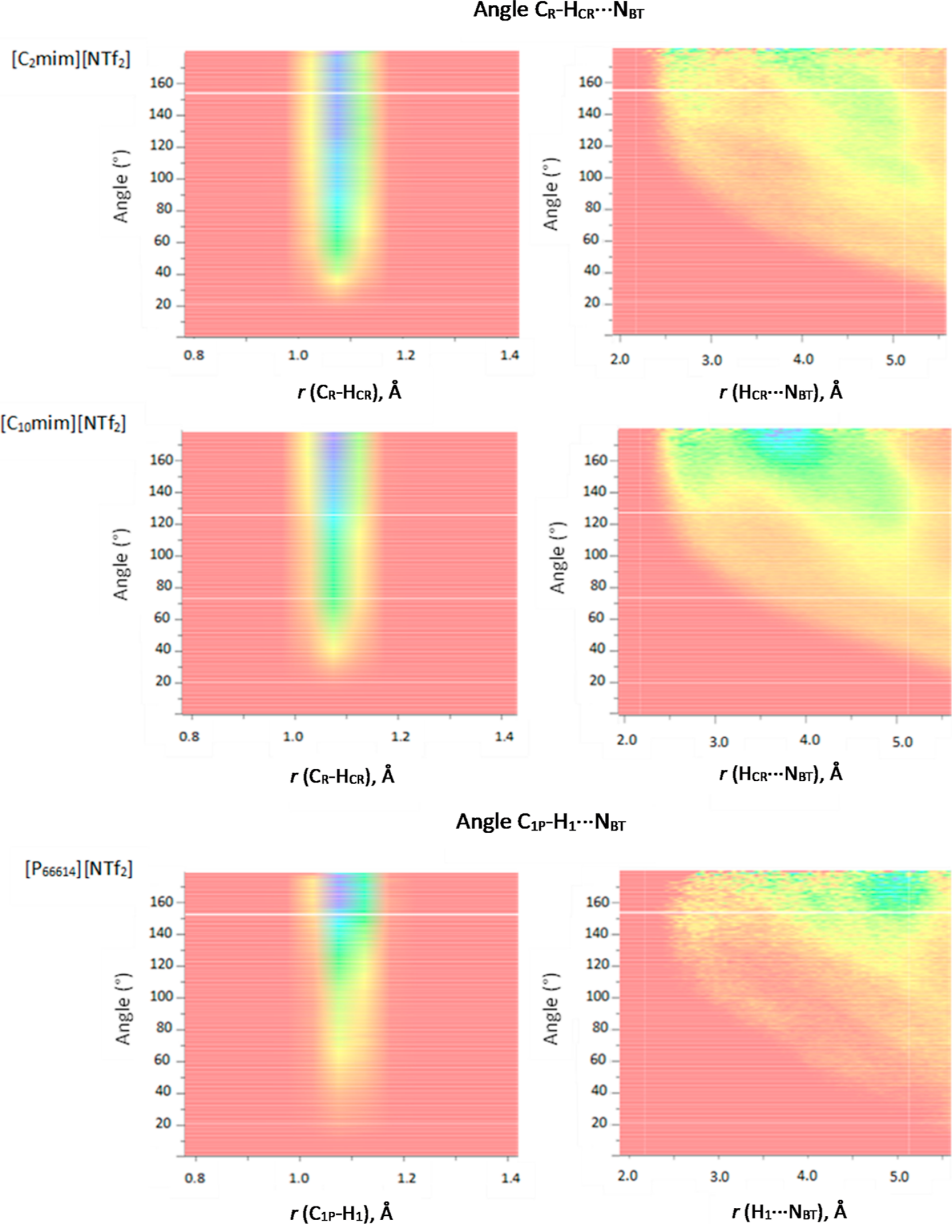
Distance and angle analysis of hydrogen bonds between atom types C_R_–H_CR_...N_BT_ for [C_2_mim][NTf_2_] and [C_10_mim][NTf_2_] and C_1P_–H_1_...N_BT_ for [P_666,14_][NTf_2_].

Dissolve provides a new capability to depict distance and angle analyses as heat maps, also known as combined distribution functions (CDFs). To the best of our knowledge, this article reports on the first CDF analysis for this set of ILs. The most interesting aspect of CDF capability was the analysis of distances and angles in C_R_–H_CR_...O_BT_ and C_1P_–H_1_...O_BT_, as well as C_R_–H_CR_...N_BT_ and C_1P_–H_1_...N_BT_, as it gives further insight into cation–anion interactions. The CDFs of [NTf_2_]^−^ hydrogen bonding motifs via oxygen, for all three ILs, are shown in Figure 9, and CDFs of [NTf_2_]^−^ hydrogen bonding motifs via nitrogen are shown in Figure 10. In Figure 9, images on the left show the C–H bond length (∼1.09 Å) on the *x* axis and the angle between this bond and the H...O hydrogen bond on the *y* axis. CDFs on the right show the reverse: the H...O hydrogen bond length (∼2.7 Å) on the *x* axis and the angle between this bond and the C–H bond on the *y* axis. Analogous representations of nitrogen hydrogen bonding are shown in Figure 10.

Despite very similar results from numerical analysis presented above, CDFs show marked differences in the interaction of [NTf_2_]^−^ anion with H_CR_ in [C_*n*_mim]^+^, when compared to its interaction with H_1_ in [P_666,14_]^+^; whereas all C–H distances are narrowly distributed around ∼1.09 Å, the angles of hydrogen bonding differ. The angles in C_R_–H_CR_...O_BT_ (in imidazolium ILs) vary between 60 and 180°, with a well-defined maximum around 130°. In contrast, the range of C_1P_–H_1_...O_BT_ angles is much more diffuse, with values starting from about 80° but the highest probability values stretching from 140 to 180°, suggesting that the C_1P_–H_1_...O_BT_ interaction is much more linear than that of C_R_–H_CR_...O_BT_. Hydrogen bonds to the nitrogen of [NTf_2_]^−^ increase in linearity as the bulk of the cation increases, which probably results from steric hindrance around the hydrogen bond donor sites (Figure 10). Comparing CDFs for H...O distances (Figure 9, right) and H...N distances (Figure 10, right), the interactions with nitrogen are less directional, with distance distribution further diffused along the *x* axis. Conventionally, more linear hydrogen bonds are stronger;^72^ these results raise an interesting question: whether hydrogen bonding between [NTf_2_]^−^ and H_1_ protons in [P_666,14_]^+^ is indeed stronger than that between the ring protons in [C_*n*_mim]^+^.

### Model Robustness

The robustness of Dissolve analysis was verified by comparative simulations using three different sets of atomic charges on the cation, sourced from ESP, LPG, and CLP force fields (Tables S3–S5). Figures S38–S40 show the comparisons of experimental and simulated total structure factors, *F*(*R*), and the corresponding Fourier transforms to real space, *G*(*r*), for the three ILs, with the three sets of potential charges applied. The quality of fit to the experimental data and the comparisons of fits between the three sets of charges are very similar. The residual *R*-factor values are also very close, and at least of the order of 10^–4^, which represents an excellent agreement between the three models and the experiment.

### Center-of-Mass Radial Distribution Functions

**Figure 11:**
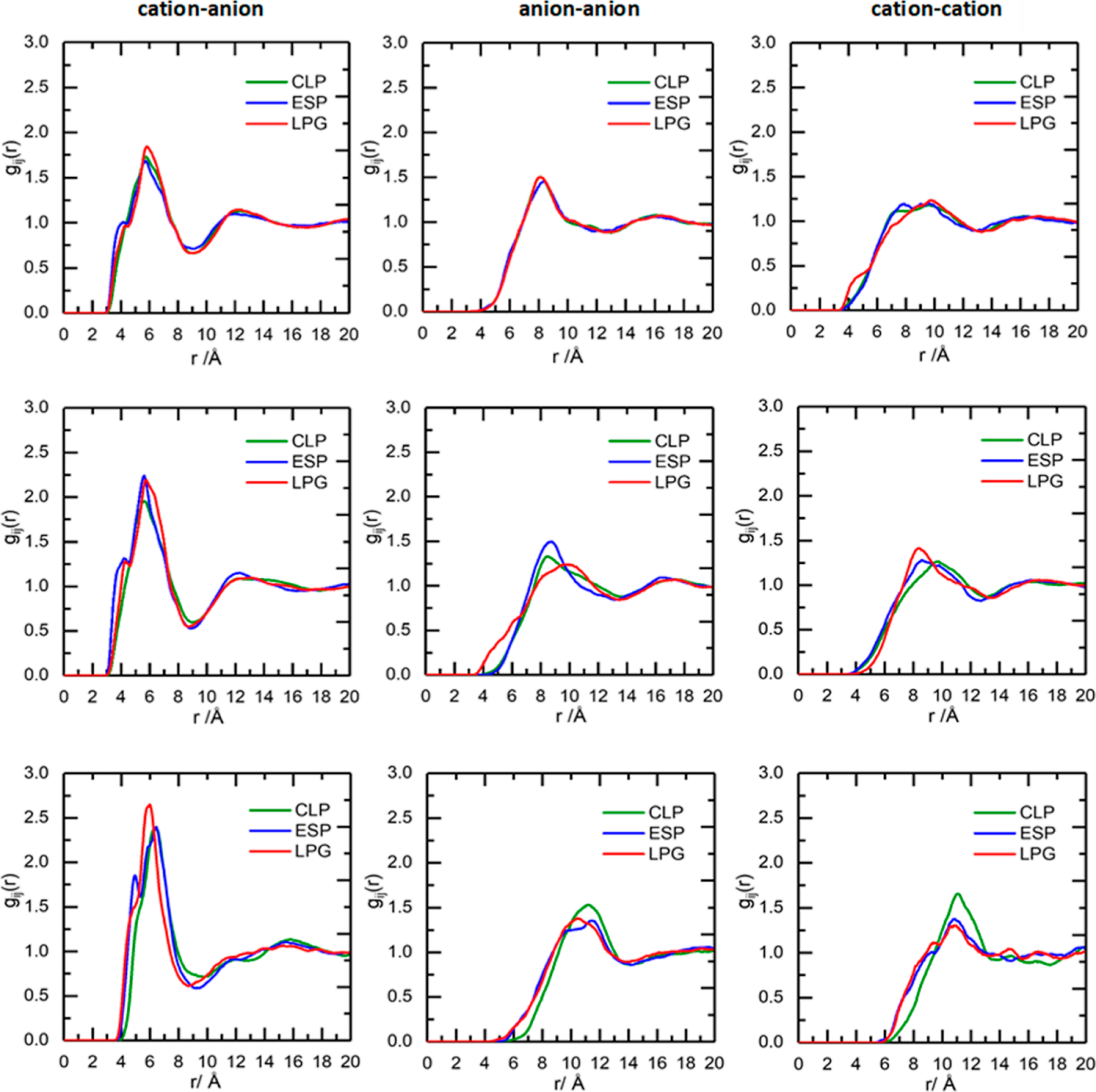
RDFs for top: [C_2_mim][NTf_2_], middle: [C_10_mim][NTf_2_], and bottom: [P_666,14_][NTf_2_] for cation–anion (left), anion–anion (middle), and cation–cation (right) interactions for CLP (green line), ESP (blue line), and LPG (red line) charges.

The RDFs for the three main interactions, cation–anion, anion–anion, and cation–cation interactions, are shown in Figure 11 for each of the three sets of charges for the three ILs. The cation–anion RDFs are similar across the three sets of charges, with a peak centered at 6 Å and a first minimum at 9 Å. The only difference, which is consistent across all three ILs, arises from the double peak for the first-shell correlation at around 5 and 6 Å, which has been attributed earlier to *cis* and *trans* [NTf_2_]^−^ conformers.^13,30,31,66^ The peak at 5 Å is more pronounced with ESP (blue line in Figure 11) and LPG (red line in Figure 11) charges for all three ILs. The first shell cation–anion CNs are in good agreement across the three charge sets for all ILs.

The anion–anion RDFs are similar upon changing cation atomic charges for both [C_2_mim][NTf_2_] and [P_666,14_][NTf_2_]. They are also very similar for [C_10_mim][NTf_2_], but the use of LPG charges resulted in a slight shift in the peak maximum from 9 to 10 Å and a shoulder preceding the peak, which is not observed with the two other charge sets.

The cation–cation RDFs for [C_2_mim][NTf_2_] are also very similar, all three models returning broad peaks between 7 and 10 Å and a minimum at 13 Å. The most obvious difference is the shoulder at 4 Å for LPG charges. Again, the profiles are very similar for [C_10_mim][NTf_2_], with just a slight shift in the peak maximum with the CLP charges. For [P_666,14_][NTf_2_], the peak maximum is consistent across the three charge sets, but a more defined shoulder preceding this peak is observed with the ESP and LPG charges compared to CLP charges. Upon changing the cation atomic charges, similar profiles are generated across the three interactions for all the ILs. Despite minor differences, all three potential charges gave convergent results, which confirms the robustness of the Dissolve analysis.

### Cis/Trans Behavior of the [NTf_2_]^−^ Anion

**Figure 12:**
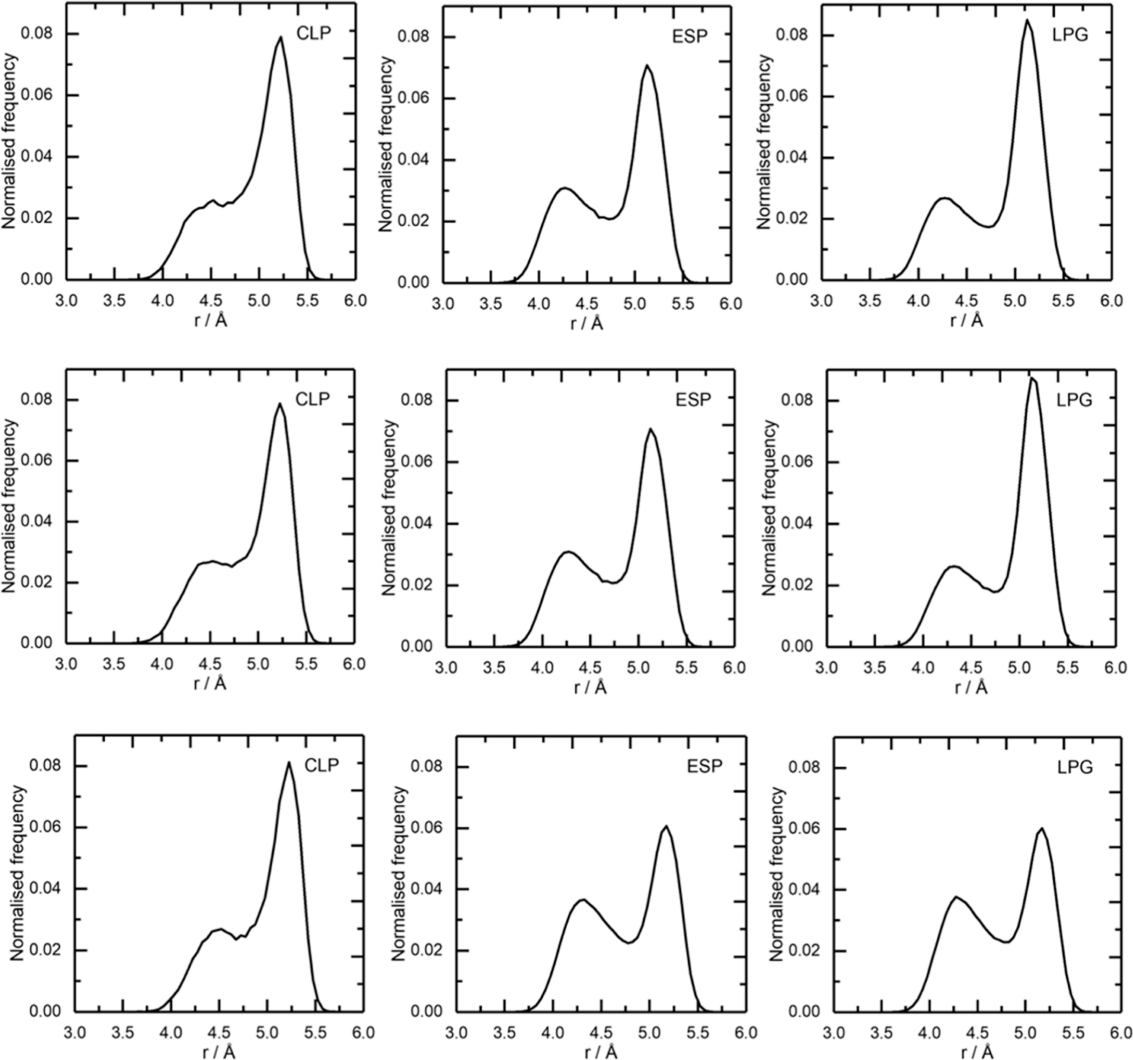
Distribution of the CF_3_...CF_3_ distances in the bis(trifluoromethanesulfonyl)amide anion, showing the cis and trans configurations in [C_2_mim][NTf_2_] (top), [C_10_mim][NTf_2_] (middle), and [P_666,14_][NTf_2_] (bottom) for the three sets of charges.

The [NTf_2_]^−^ anion can adopt both *cis* and *trans* orientations in the liquid state. The *cis*/*trans* ratio is commonly obtained from the distribution of CF_3_...CF_3_ distance, which was determined for each IL across the three different charges sets (Figure 12). An intramolecular CF_3_...CF_3_ distance is 4.2 Å for the *cis* orientation and 5.2 Å for the *trans* orientation. It has been found that there is greater distribution of the *trans* conformer in all three ILs, in agreement with both computational and experimental studies on [NTf_2_]^−^ ILs, particularly in the multitude of Raman spectroscopic studies.^29,66,68,73^ The preference for the *trans* conformer can be explained due to the greater availability of this orientation to form hydrogen bonds and the reduction of steric repulsion.

## Conclusions

In conclusion, the present study works toward removing some barriers in the study of bulky ILs, and other amorphous organic materials, by neutron scattering. To remove the first barrier, a detailed method for the synthesis of fully deuterated [P_666,14_][NTf_2_] is reported, hopefully enabling enhanced neutron scattering studies of tetraalkylphosphonium ILs. Furthermore, in addition to [C_2_mim][NTf_2_], the structure of two ILs with long alkyl chains, [C_10_mim][NTf_2_] and [P_666,14_][NTf_2_], has been resolved for the first time using neutron scattering, enabled by the new Dissolve data analysis package. Robustness of the Dissolve approach has been demonstrated by generating three independent models for each of the three ILs, starting from three different potential sets for cations and reaching convergent results for each IL, across the three models. Furthermore, general structural features observed experimentally match those predicted by published MD simulations. Thereby, the combination of Dissolve data analysis package and synthetic developments enabling contrast from deuteration aid to bridge the gap between insights from computational simulations and experimental studies.

Analysis of the neutron scattering data showed that [C_10_mim][NTf_2_] and [P_666,14_][NTf_2_] exhibit substantial nanosegregation compared to that of [C_2_mim][NTf_2_], induced by the presence of long alkyl chains. It has been demonstrated that protons on the imidazolium ring in [C_2_mim][NTf_2_] and [C_10_mim][NTf_2_], as well as H–C–P protons in [P_666,14_][NTf_2_], participate in hydrogen bonding, with oxygen and nitrogen atoms in the [NTf_2_]^−^ anion acting as hydrogen bond acceptors, with the dominant interaction to the oxygen. From bond distance and angle analysis, it was evident that bulkier cations promote more linear hydrogen bonds and that hydrogen bonding to oxygen is more directional than that to nitrogen. Since more linear hydrogen bonds are considered to be stronger, this inspires a question whether hydrogen bonding between [NTf_2_]^−^ and H_1_ protons in [P_666,14_]^+^ is indeed stronger than that between the ring protons in [C_*n*_mim]^+^.

## Supporting Information Available

Additional experimental details, NMR spectra of all compounds, and details of Dissolve simulation parameters and fits (PDF)
