# Supplementary material for: Liquid Structure of Ionic Liquids with [NTf2]− Anions, Derived from Neutron Scattering
Source: J Phys Chem B. 2024 Mar 23;128(13):3220–35. doi: 10.1021/acs.jpcb.3c08069 (PMC11000221; doi:10.1021/acs.jpcb.3c08069)
Supplement: Supplementary file 1 — jp3c08069_si_001.pdf [file jp3c08069_si_001.pdf]

The Liquid Structure of Ionic Liquids with $[NTf_2]^-$ Anions, Derived From Neutron Scattering

Anne McGrogan,[a] Jack Lafferty,[a] Lauren O'Neill,[a], Lucy Brown,[a] J. Mark Young,[a] Peter Goodrich,[a] Mark J. Muldoon,[a] Leila Moura,[a] Sarah Youngs,[b] Terri-Louise Hughes,[b] Sabrina Gärtner,[b] Tristan Youngs,[b] John D. Holbrey*[a] and Małgorzata Swadźba-Kwaśny*[a]*

[a] QUILL Research Centre, Queen's University Belfast, School of Chemistry and Chemical Engineering, David Keir Building, 39-123 Stranmillis Road, Belfast, BT9 5AG, Belfast, UK

[b] Rutherford Appleton Laboratory, Chilton, Didcot, OX11 0QX, UK

Materials

Trihexyl(tetradecyl)phosphonium chloride, $[P_{666,14}]Cl$, was kindly provided by Solvay. Perdeuteriated 1-hexanol and 1-tetradecanol as well as perdeuteriated $[C_2mim][NTf_2]$ and $[C_{10}mim][NTf_2]$ were provided from the deuteration facility at ISIS Neutron and Muon source. Unless otherwise stated, all other chemicals were purchased from Sigma-Aldrich and used as received. XRF analysis was performed on a Rigaku NEX QC+ QuantEZ High-Resolution Energy Dispersive X-ray Fluorescence (EDXRF) Spectrometer. NMR spectra were recorded on either a Bruker Avance III 400 MHz spectrometer or a Bruker Avance II DPX 600 MHz spectrometer. Quantitative ^1H NMR was recorded on a Bruker Avance III 400 MHz spectrometer, with benzene as an internal standard and CD_3OD as NMR solvent.

Safety

In the case of deuteration of P_{666} and $[P_{666,14}]Cl$, Pd/C and/or Pt/C catalysts are used. These catalysts are highly flammable (particularly after the reaction) and proper protocols and precautions should be used to avoid any potential fires. This involves keeping the catalyst waste wet and safe disposal after the reaction.

Synthesis of ionic liquids

$[C_2mim][NTf_2]$. $[C_2mim]Cl$ (0.027 mol, 4.00 g) and $Li[NTf_2]$ (0.030 mol, 8.59 g) were separately dissolved in water (total 30 mL) and then combined, resulting in the formation of a separate liquid phase. This was left to stir for 1 h at room temperature. The aqueous layer was separated out and the organic layer was collected and washed. The first wash was with 10 mL of deionised water and 10 mL of

dichloromethane (DCM). Subsequent washes (7) were performed with deionised water. Two subsequent negative tests for chloride with $AgNO_3$ were required to ensure removal of LiCl. DCM was removed *via* reduced pressure at 35 °C, and the product was a colourless viscous ionic liquid. The ionic liquid was dried overnight on the Schlenk line at a temperature of 100 °C. ^{1}H NMR: (400 MHz, d_6 -DMSO) δ 1.43 (t, 3H), 3.84 (s, 3H), 4.18 (t, 2H), 7.68 (t, 1H), 7.76 (t, 1H), 9.09 (s, 1H). ^{13}C NMR (400 MHz, d_6 -DMSO) δ 14.98, 35.66, 44.20, 119.94 (q, $^{1}J_{C-F}$ = 1280 Hz CF_3), 121.97, 123.58, 136.28. ^{19}F NMR (400 MHz, d_6 -DMSO) δ -79.82

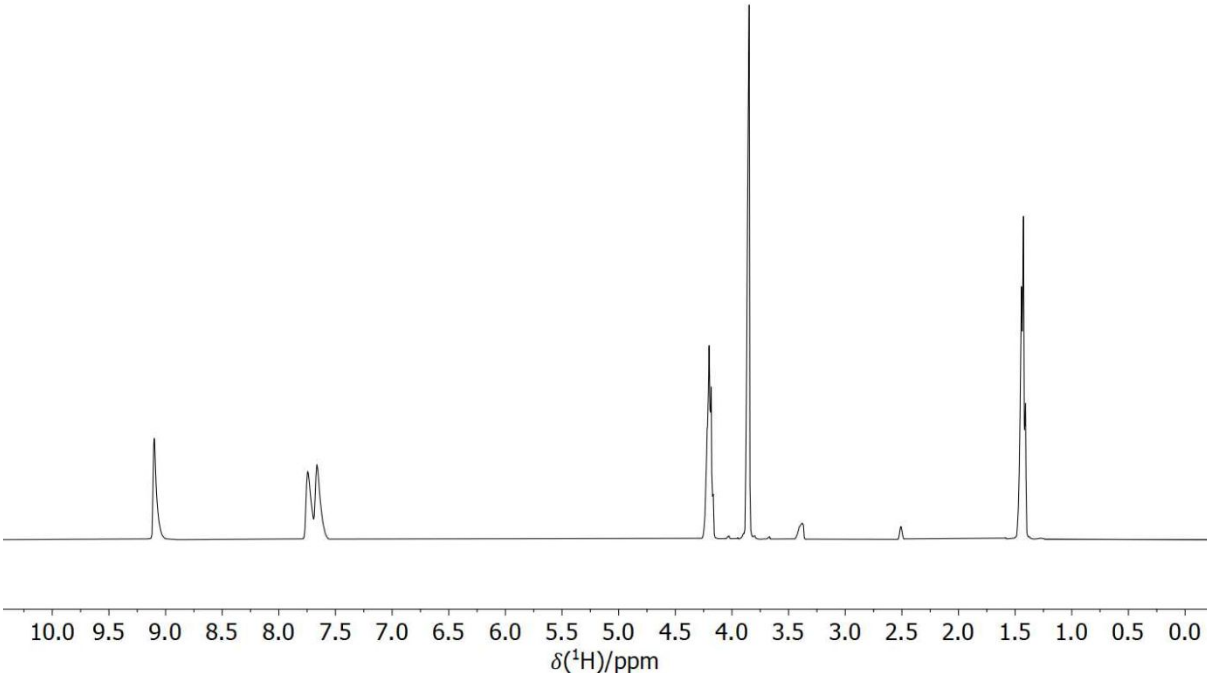

Figure S1. ^{1}H NMR of $[C_2mim][NTf_2]$ in d_6-DMSO.

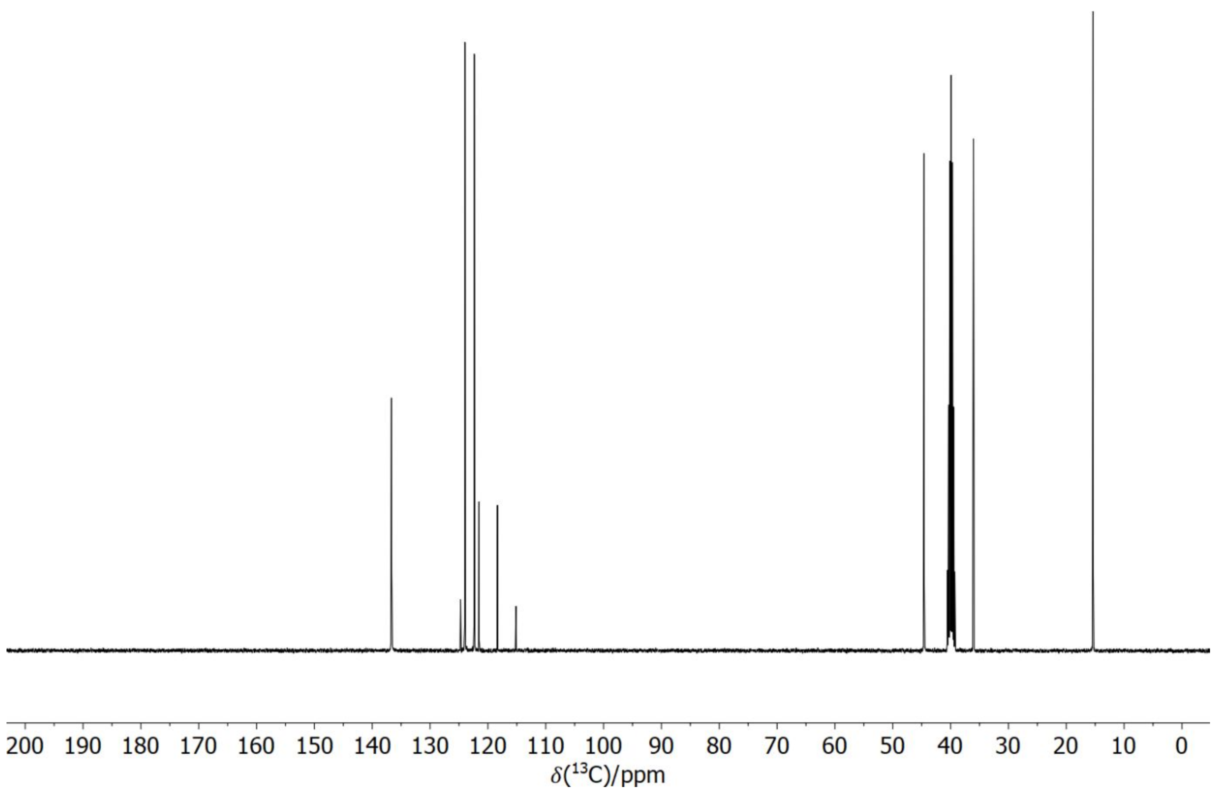

Figure S2. ^{13}C NMR of $[C_2mim][NTf_2]$ in d_6-DMSO

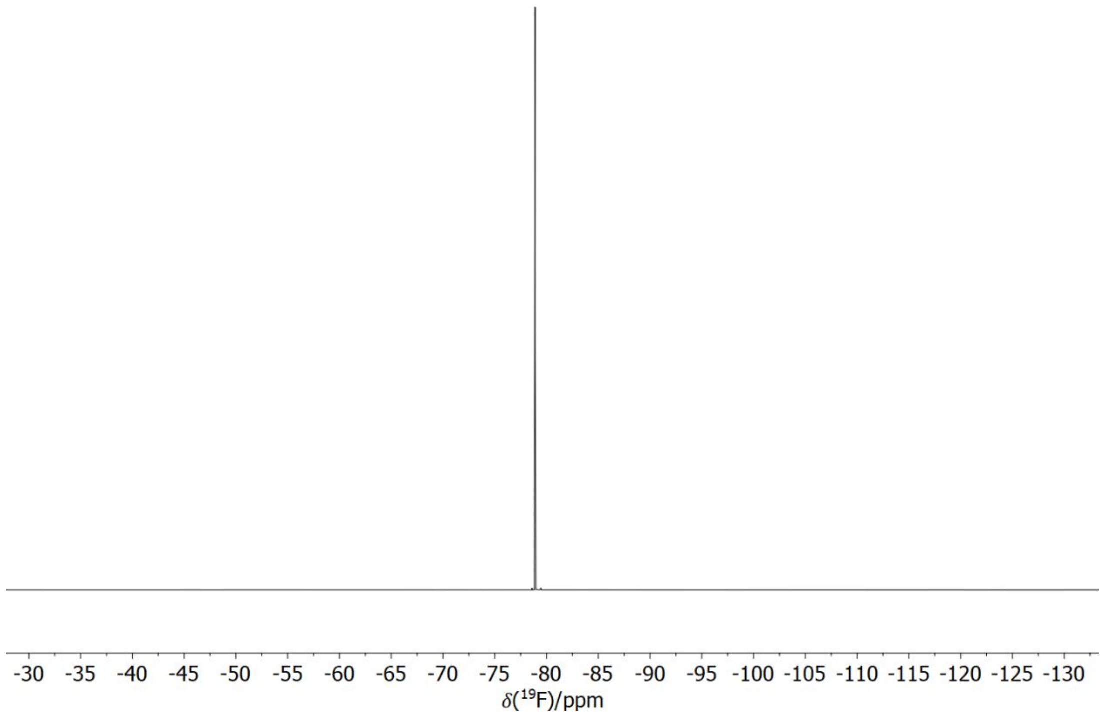

Figure S3. ^{19}F NMR of $[C_2mim][NTf_2]$ in d_6-DMSO.

[C_{10}mim][NTf_2]. [C_{10}mim]Br (0.073 mol, 22.11 g) and Li[NTf_2] (0.080 mol, 23.03 g) were separately dissolved in water (total 30 ml) and then combined, resulting in the formation of a separate liquid phase. This was left to stir for 1 h at room temperature. The aqueous layer was separated out and the organic layer was collected and washed. The first wash was with 10 mL of deionised water and 10 mL of dichloromethane (DCM). Subsequent washes (7) were performed with deionised water. Two subsequent negative tests for chloride with $AgNO_3$ were required to ensure removal of LiCl. DCM was removed *via* reduced pressure at 35 °C, and the product was a colourless viscous liquid. The ionic liquid was dried overnight on the Schlenk line at a temperature of 100 °C. ^{1}H NMR (400 MHz, d_6 -DMSO): 8.72 (s, 1H), 7.58 (t, 1H), 7.50 (t, 1H), 4.28 (m, 2H), 4.00 (s, 3H), 1.99 (m, 2H), 1.39 (m, 14H), 0.98 (t, 3H). ^{13}C NMR (400 MHz, d_6-DMSO) δ 136.94, 122.65, 124.75, 119.35 (q, $^1J_{C\text{-}F}$ = 1276 Hz CF_3), 49.27, 36.09, 31.73, 29.84, 29.34, 29.25, 29.11, 28.81, 25.93, 22.51, 14.19. ^{19}F NMR (400 MHz, d_6 -DMSO) δ -80.12

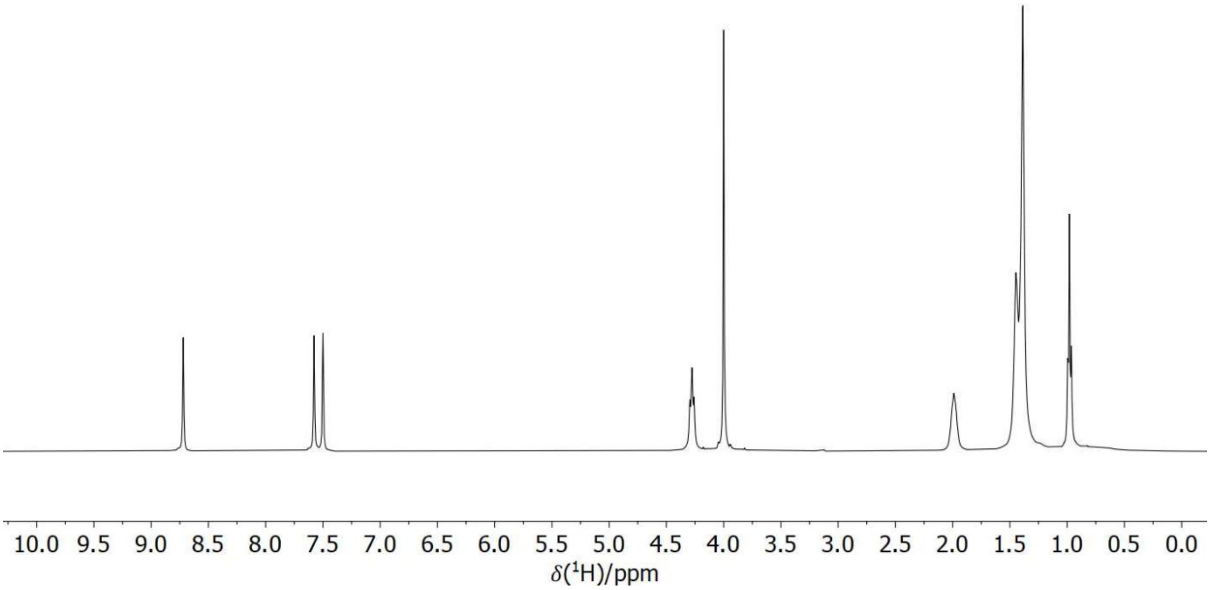

Figure S4. ^{1}H NMR of [C_{10}mim][NTf_2] with a DMSO-d_6 capillary.

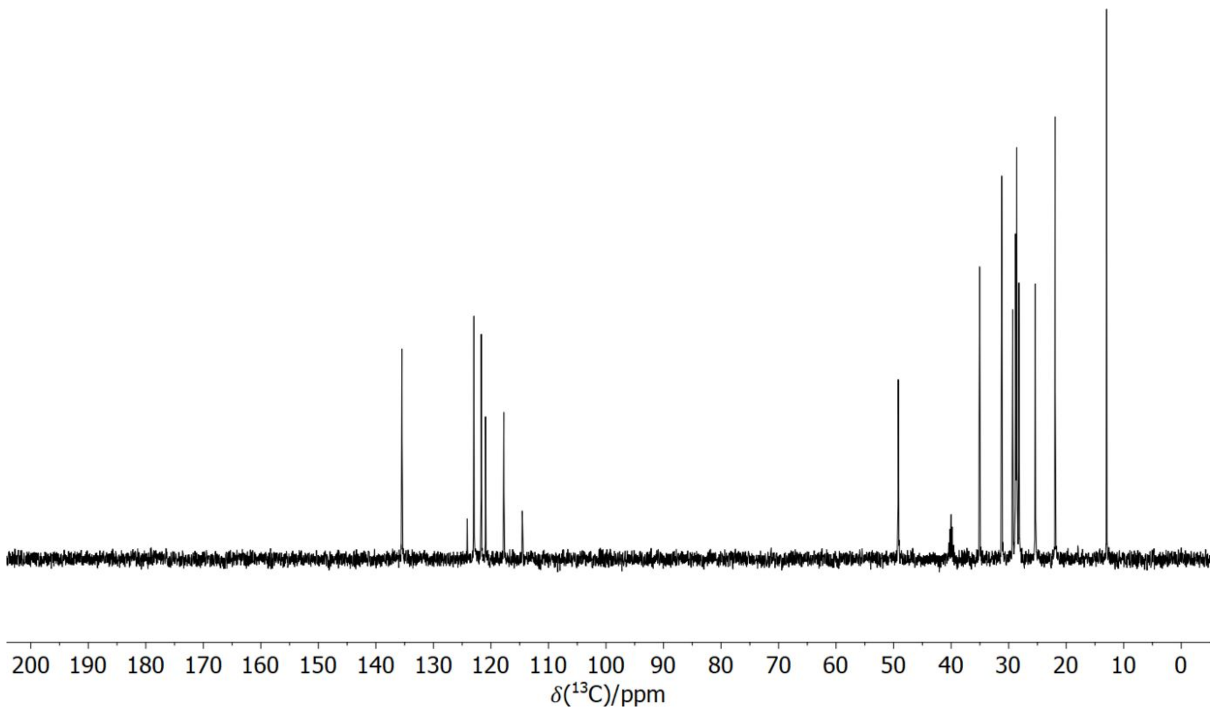

Figure S5. ^{13}C NMR of [C_{10}mim][NTf_2] with a DMSO-d_6 capillary.

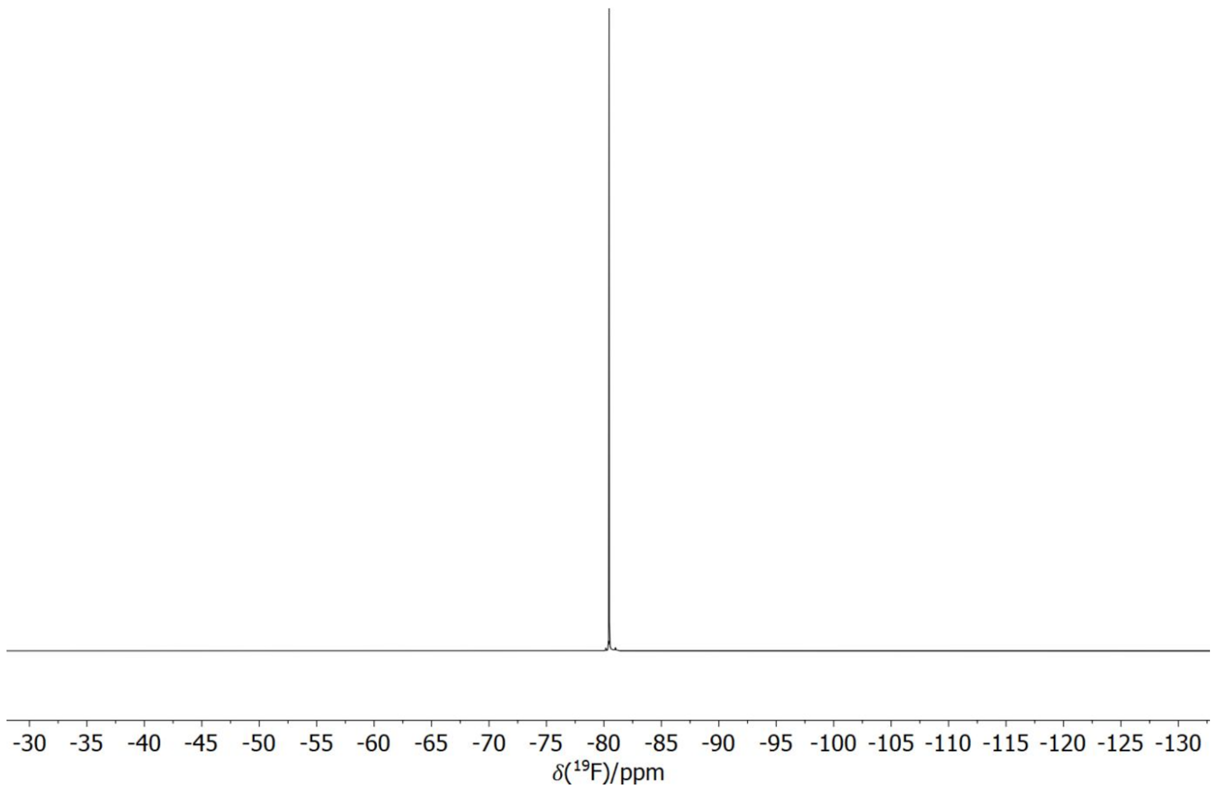

Figure S6. ^{19}F NMR of [C_{10}mim][NTf_2] with a DMSO-d_6 capillary.

[$P_{666,14}$][NTf_2]. Trihexyl(tetradecyl)phosphonium chloride, [$P_{666,14}$]Cl (0.010 mol eq.) and lithium bis(trifluoromethanesulfon)imide Li[NTf_2] (0.013 mol eq.) were separately dissolved in 25 mL deionised water (total 50 mL) and then combined resulting in the formation of a biphasic liquid system; the mixture was left to stir for 1 h at room temperature, 600 rpm. The aqueous layer was separated, and the organic layer was collected and washed, firstly with deionized water (10 mL) and then dichloromethane, DCM (10 mL). Subsequent washes were performed with solution of Li[NTf_2] in deionised water. Final three washes were performed with deionised water until no chloride could be detected with silver nitrate solution. Subsequently, DCM was removed *via* rotary evaporation and the ionic liquid was dried under high vacuum (12 h, 70 °C, 10^{-2} mbar). XRF analysis confirmed chloride content was below the detectable limit. ^{1}H, ^{13}C, ^{19}F and ^{31}P NMR spectra of the IL were recorded in $CDCl_3$. ^{1}H NMR (400 MHz, $CDCl_3$) δ 0.89 (m, 12H), 1.26-1.48 (m, 48H), 2.11 (m, 8H). ^{13}C NMR (400 MHz, $CDCl_3$) δ: 13.53, 13.64, 17.45, 17.52, 20.46, 20.50, 20.54, 21.75, 22.07, 28.81, 28.90, 29.04, 29.09, 29.70, 29.95, 30.34, 31.32, 119.50 (q, $^{1}J_{C-F}$ = 1280 Hz CF_3). ^{19}F NMR (400 MHz, $CDCl_3$) δ -79.00. ^{31}PNMR (400 MHz, $CDCl_3$) δ 32.94.

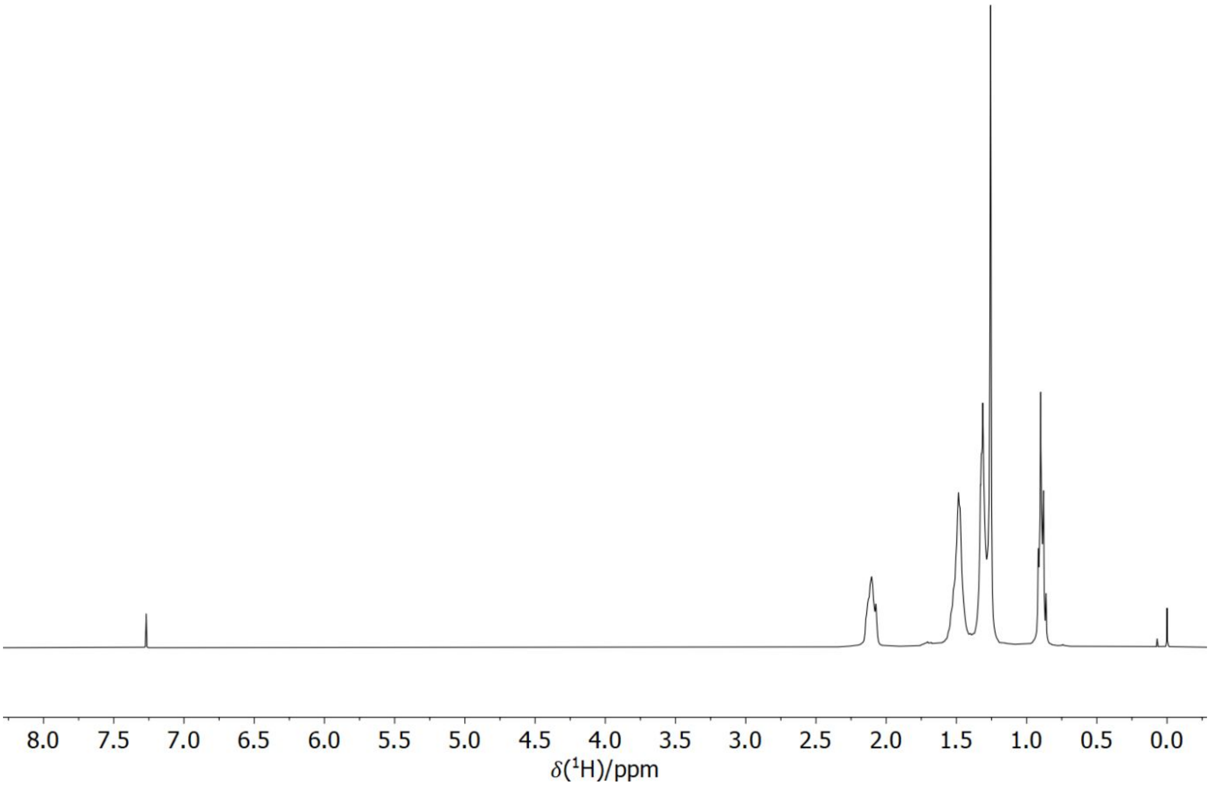

Figure S7. ^{1}H NMR of [$P_{666,14}$][NTf_2] in $CDCl_3$.

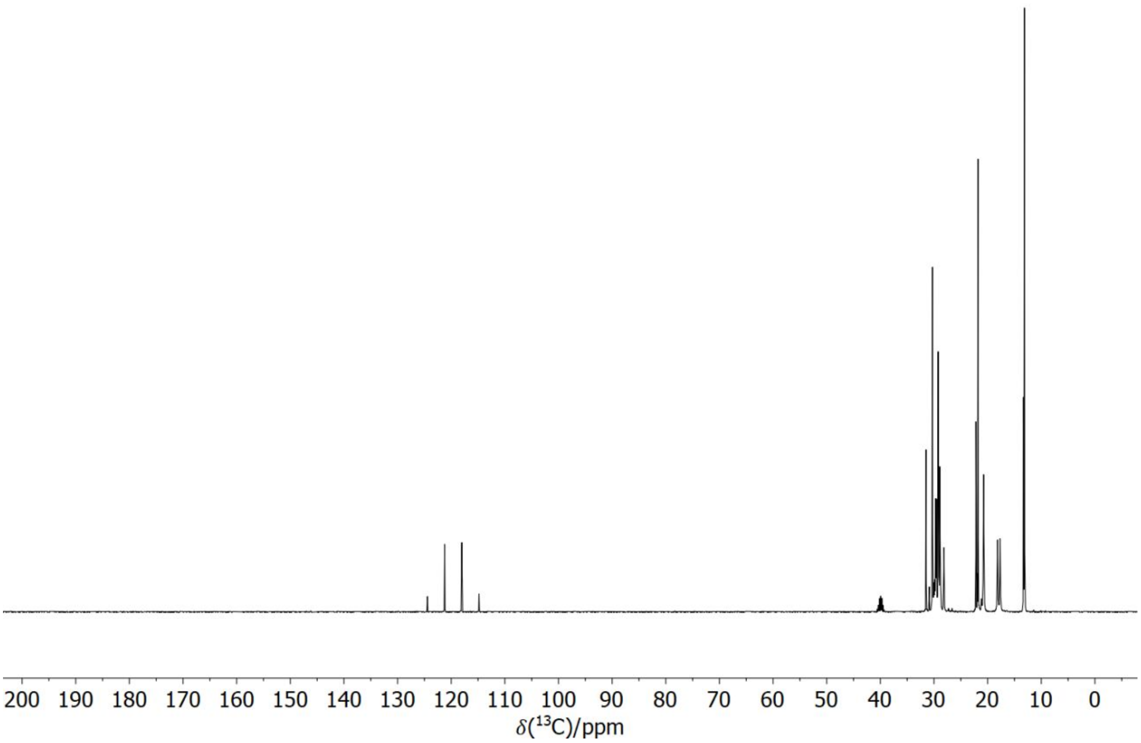

Figure S8. ^{13}C NMR of $[P_{666,14}][NTf_2]$ in $CDCl_3$

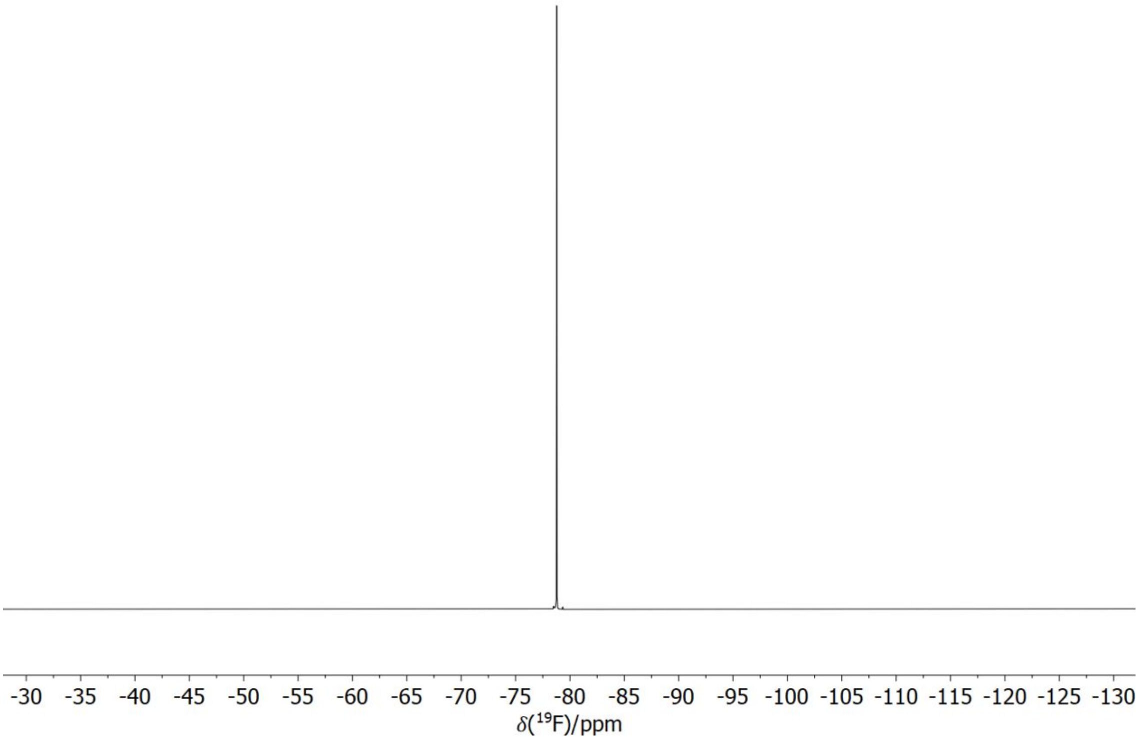

Figure S9. ^{19}F NMR of $[P_{666,14}][NTf_2]$ in $CDCl_3$

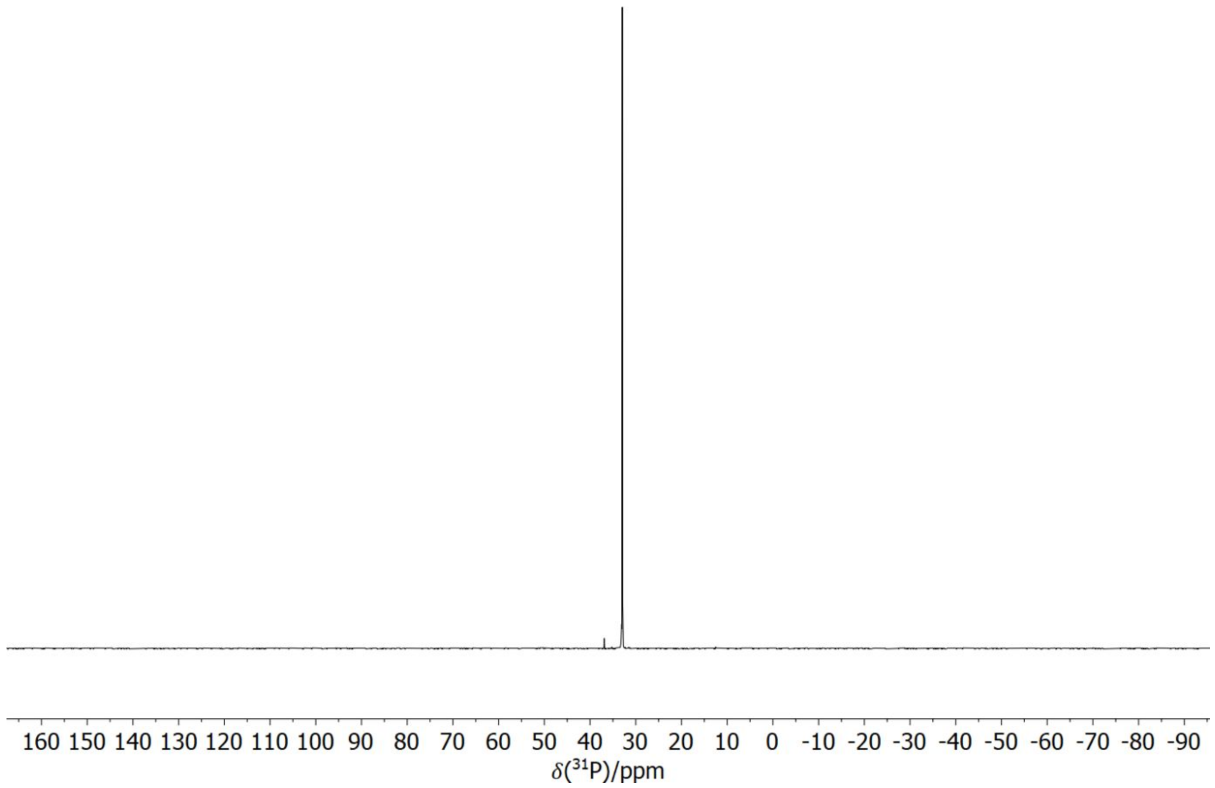

Figure S10. ^{31}P NMR of $[P_{666,14}][NTf_2]$ in $CDCl_3$

Method 1: Attempted direct deuteration of $[P_{666,14}]^+$ cation of $[P_{666,14}]Cl$

The reaction was carried out in a 100 mL Parr high pressure reactor fitted with a mechanical stirrer. 5.08 g of $[P_{666,14}]Cl$, 0.50 g NaOH, 0.25 g Pt/C and 50 ml D_2O were added to the 100 mL Parr reactor body (made of Hastelloy c276). After the reactor was sealed, the reactor was pressurised with nitrogen (40-50 bar). The reactor was then heated to 180 °C using the heating mantle and stirred at 600 RPM for 3 days. After cooling, the reaction mixture was diluted with dichloromethane, filtered through Celite, washed with dichloromethane and then the aqueous phase was extracted with dichloromethane (3 x 50 mL). Dichloromethane was removed by rotary evaporation and four subsequent cycles were performed. $[P_{666,14}][OH]$ was formed and mass spec showed that 8 hydrogens (four P–CH_2 protons) of the 68 hydrogens were deuterated.

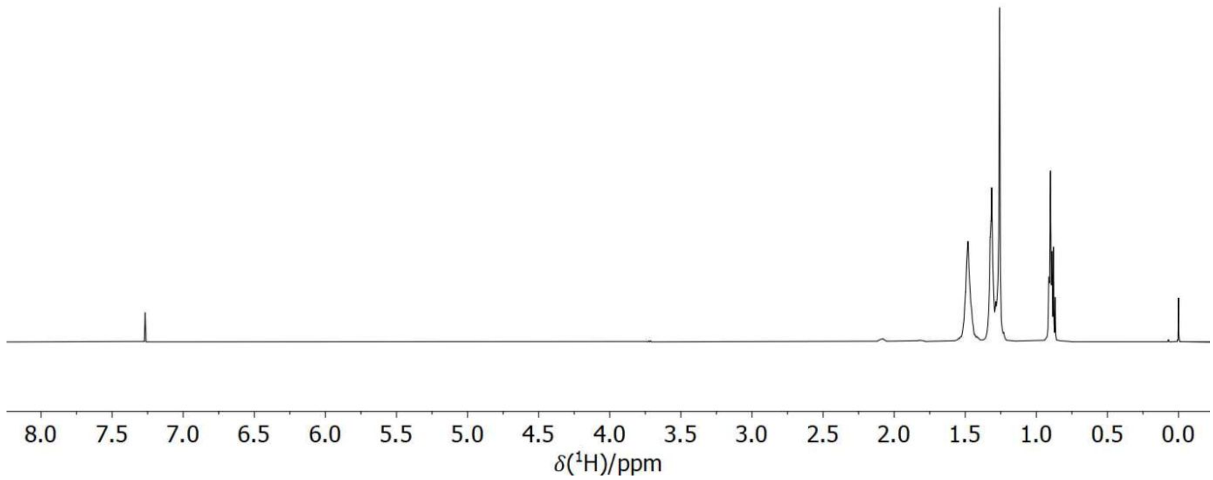

Figure S11. ^{1}H NMR of D_8-[$P_{666,14}$][NTf_2] in $CDCl_3$.

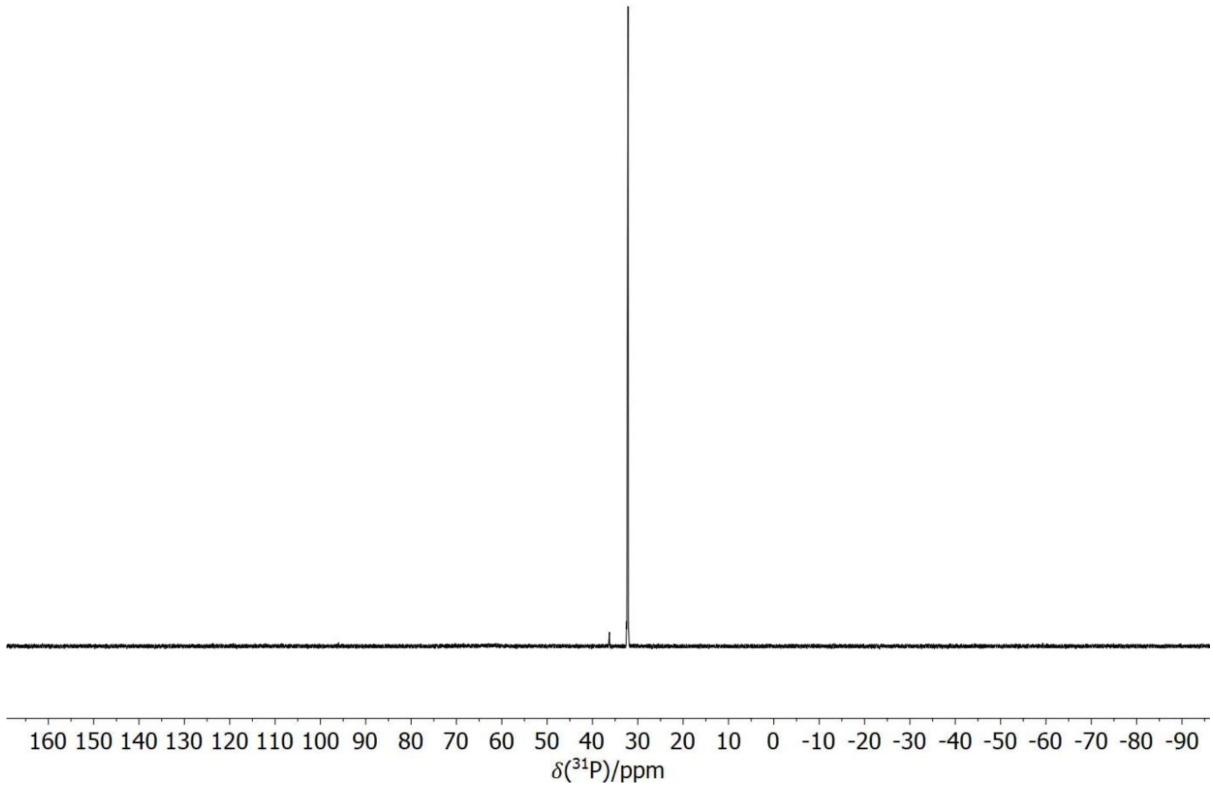

Figure S12. ^{31}P NMR of D_8-[$P_{666,14}$][NTf_2] in $CDCl_3$.

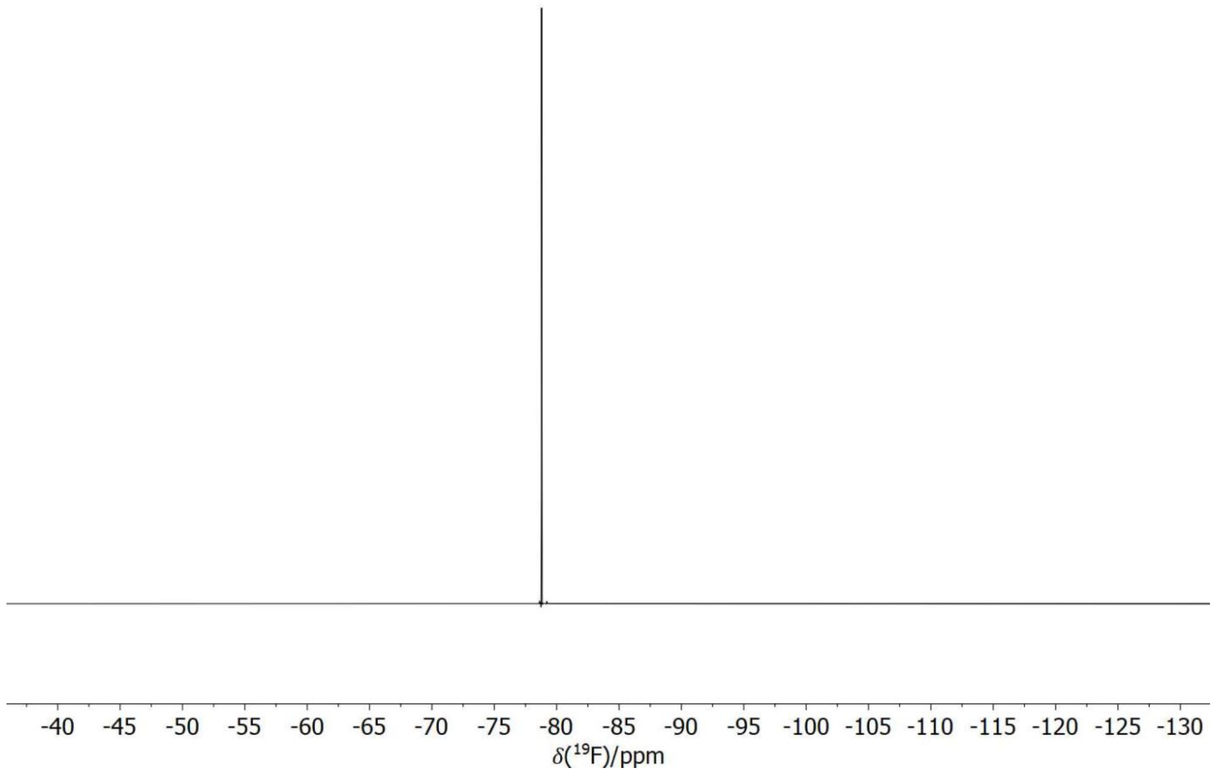

Figure S13. ^{19}F NMR of D_8-[$P_{666,14}$][NTf_2] in $CDCl_3$.

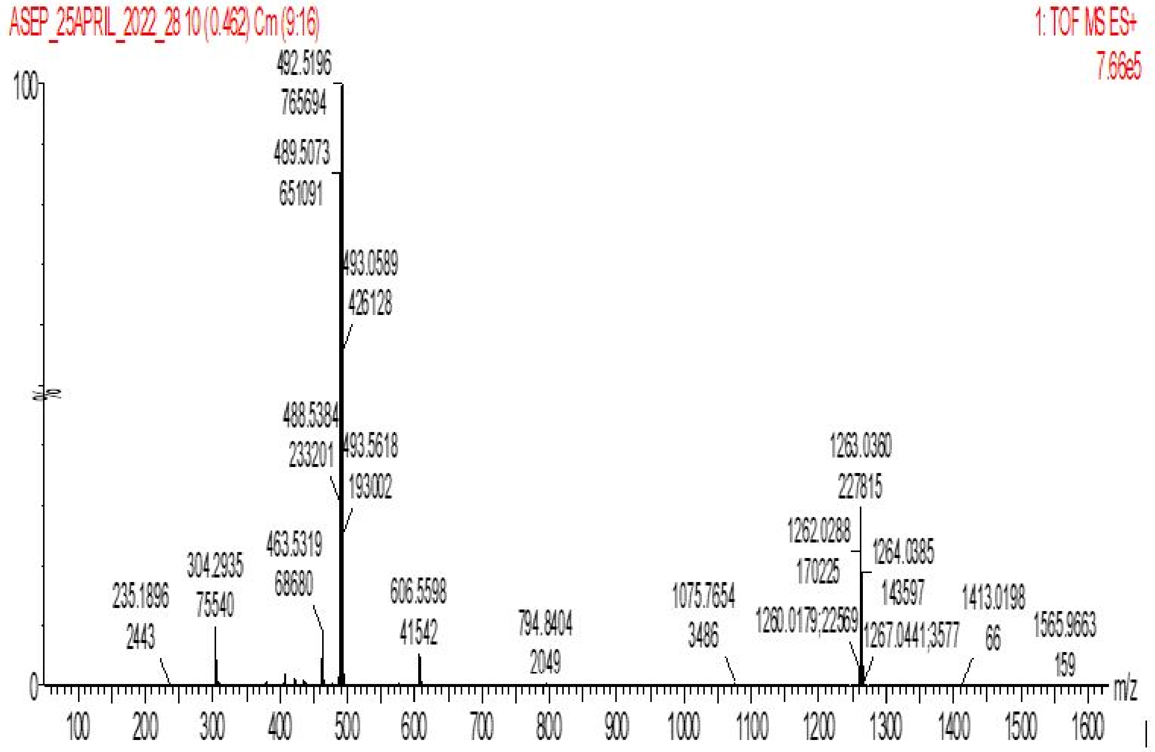

Figure S14. ESI-MS of D_8-$[P_{666,14}][NTf_2]$ in positive mode

Method 2: Attempted synthesis of D_{39}-trihexylphosphine *via* Grignard reaction

Synthesis of D_{13}-1-chlorohexane

Thionyl chloride (27.62 g, 0.232 mol) was added to a 2-necked round bottom flask equipped with a PTFE coated magnetic stirrer bar, condenser and pressure equalising dropping funnel, which were both fitted with calcium chloride guard tubes. D_{13}-hexanol (8.92 g, 0.0774 mol) was then added to the pressure equalising dropping funnel and added slowly to the thionyl chloride with stirring. As the reaction progressed, heat and SO_2 evolved. When all the alcohol was added, the mixture was heated at reflux for 2 hours. The excess of thionyl chloride was then separated from the product by distillation, (78-80 °C), with the crude D_{13}-1-chlorohexane at 132-133 °C. This was then washed with D_2O, 10% sodium carbonate solution and twice with D_2O. Then, dried with anhydrous calcium chloride and distilled again. Pure 1-chlorohexane-d_{13} passes over at 133-134 °C.^{13}C NMR (400 MHz, $CDCl_3$) δ 12.8 (m), 21.1 (m), 25.1 (m), 28.4 (m), 29.6 (m), 31.4 (m). 44.3 (m)

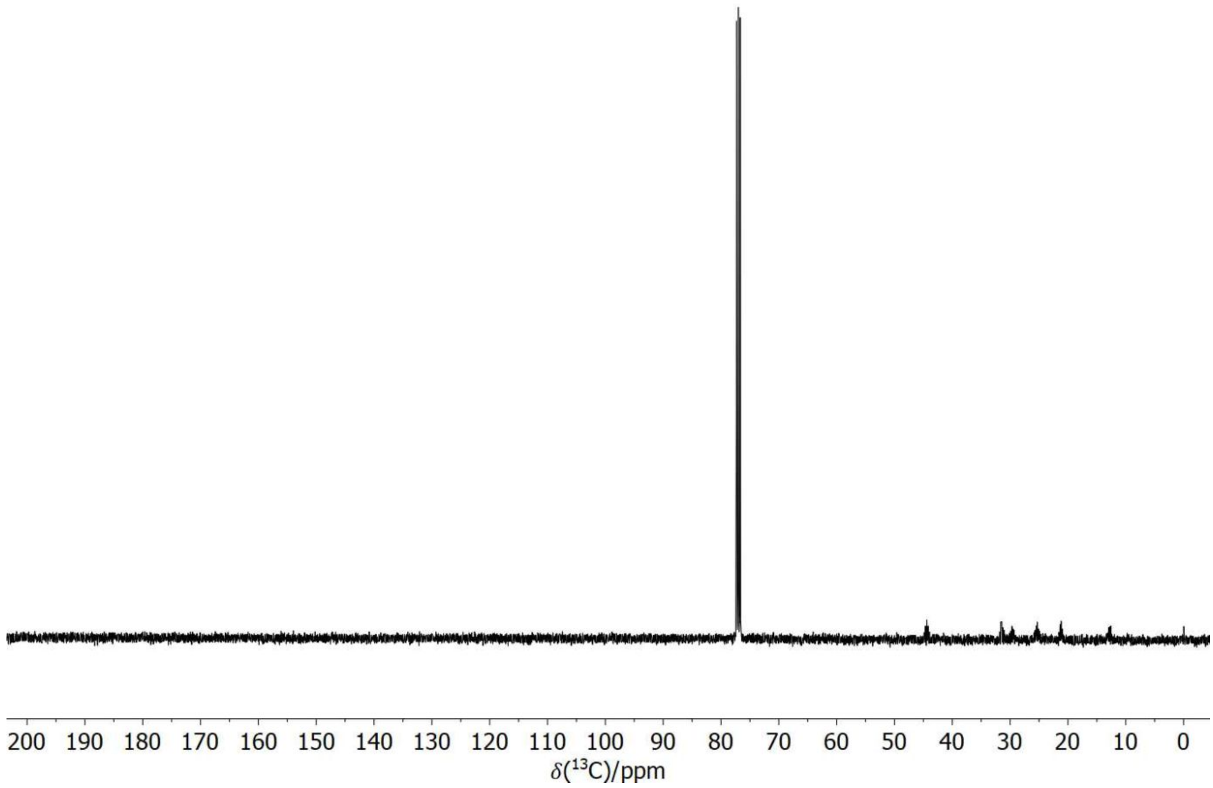

Figure S15. ^{13}C NMR of D_{13}-chlorohexane in $CDCl_3$.

Synthesis of D_{29}-1-tetradecylchloride

Thionyl chloride (13.93 g, 0.117 mol) was added to a 2-necked round-bottomed flask equipped with a PTFE coated magnetic stirrer bar, condenser, and pressure equalising dropping funnel, which were both fitted with calcium chloride guard tubes. D_{29}-tetradecanol (9.50 g, 0.0390 mol) was then added to the pressure equalising dropping funnel and added slowly to the thionyl chloride with stirring. As the reaction progressed, both heat and SO_2 evolved. When all the alcohol was added, the mixture was heated at reflux for 3 hours. The excess of thionyl chloride was then separated from the product by distillation, (78-80 °C), with the crude 1-tetradecylchloride requiring 98 °C and 0.5 mbar. This was then washed with D_2O, 10% sodium carbonate solution and twice with D_2O. Then, dried with anhydrous calcium chloride and distilled again (11.28 g, 81% yield, deuteration level 99% calculated by quantitative ^1H NMR). ^1H NMR (400 MHz, $CDCl_3$) residual protons δ 0.82 (m), 1.19 (m), 1.36 (m), 1.54 (m), 1.72 (m), 3.58 (m). ^2H NMR (400 MHz, $CDCl_3$) δ 0.85-3.48 (m). ^{13}C NMR (400 MHz, $CDCl_3$) δ 13.1 (m), 21.4 (m), 25.8 (m), 28.2 (m), 30.4 (m), 31.6 (m). 44.2 (m)

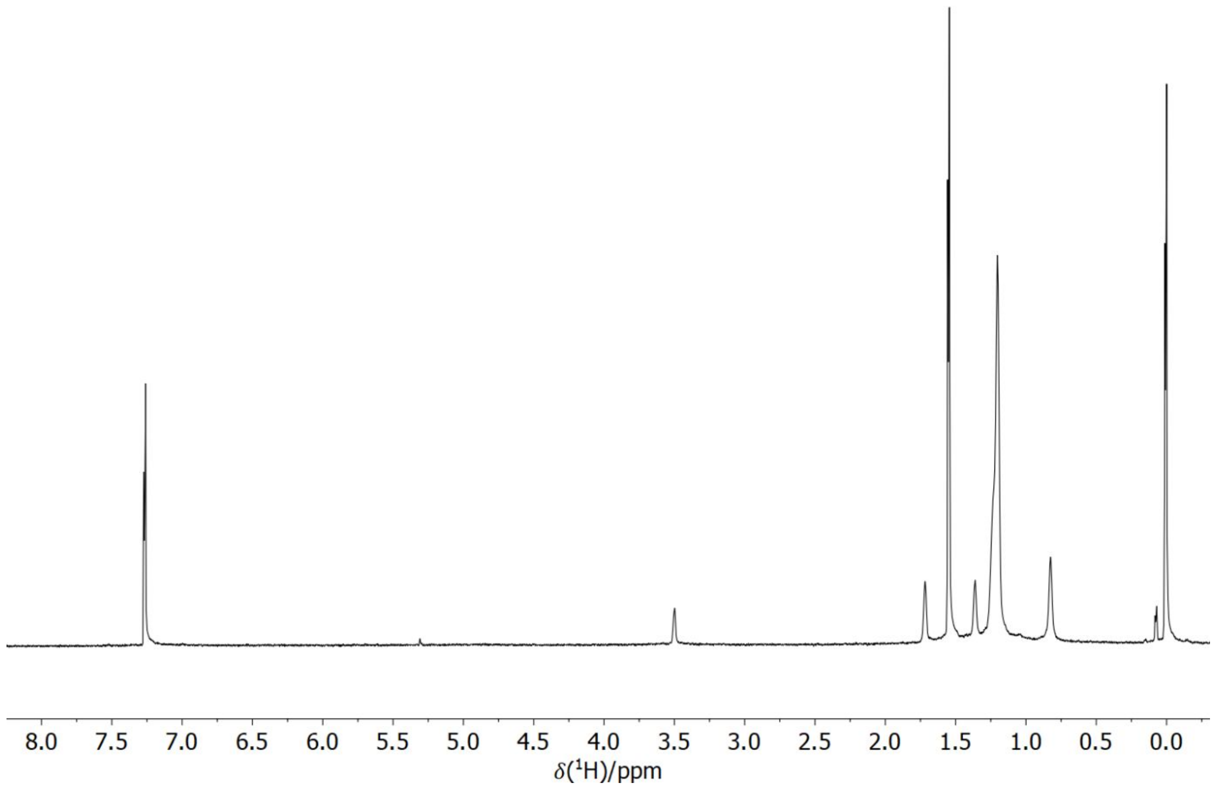

Figure S16. ^{1}H NMR of D_{29}-1-tetradecylchloride in $CDCl_3$

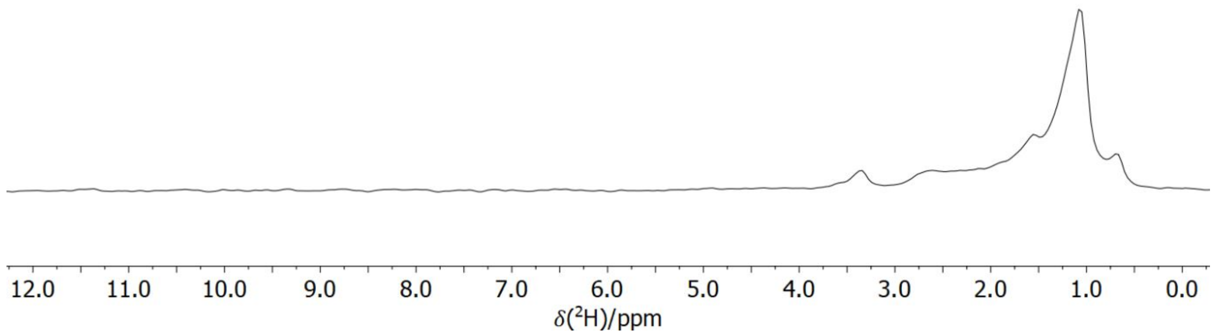

Figure S17. ^{2}H NMR of D_{29}-1-tetradecylchloride

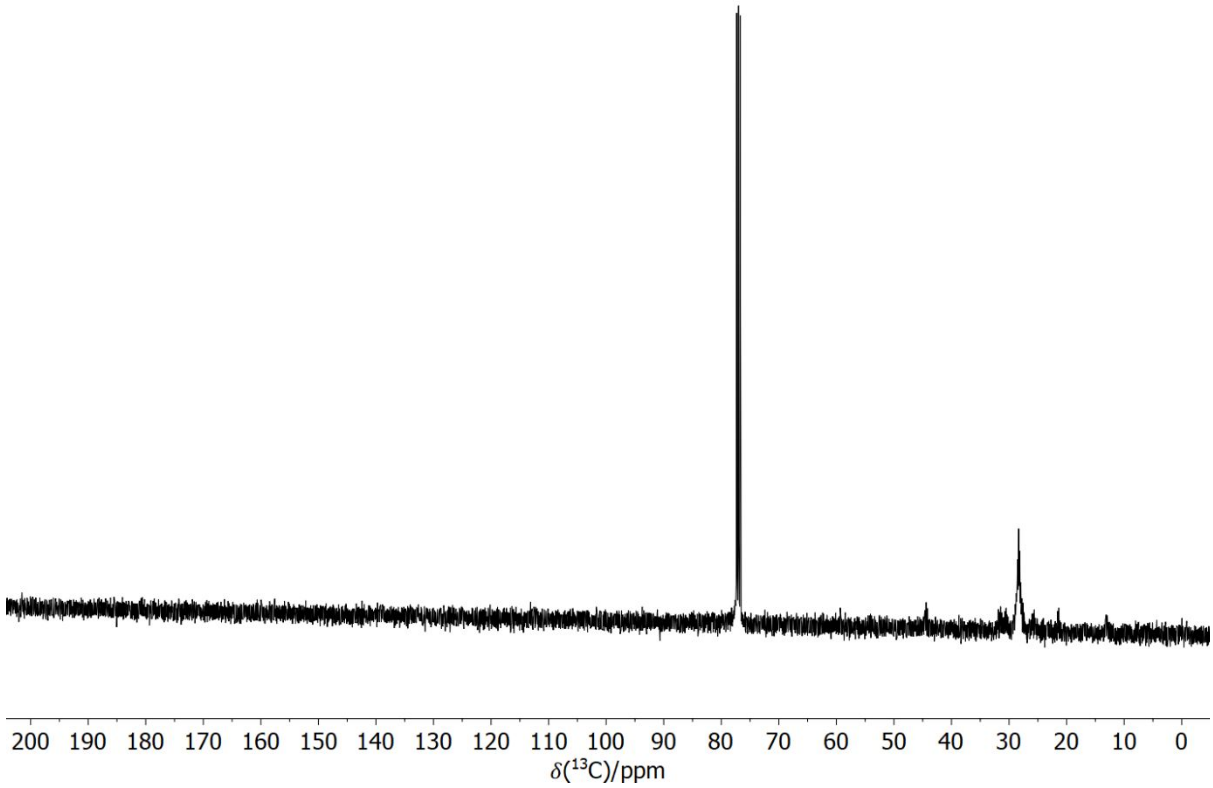

Figure S18. ^{13}C NMR of D_{29}-1-tetradecylchloride in $CDCl_3$

Attempted synthesis of D_{68}-trihexylphosphine

Magnesium turnings (0.54 g, 1.20 eq.) were transferred into an oven-dried two-necked round-bottomed flask (100 ml) equipped with a reflux condenser with argon gas inlet, a septum and a PTFE coated magnetic stirring bar. Anhydrous diethyl ether (10 cm^3) was then added, followed by a crystal of iodine, and the flask was heated to 30 °C, with stirring. Subsequently, a small portion of D_{13}-1-chlorohexane (2.51 g, 1.0 eq.) was added dropwise via a syringe. After approximately 50% was added, the yellow iodine colour disappeared, with the solution becoming a grey colour, and the commencement of gentle refluxing. The remainder of the solution was added and the reaction was allowed to proceed (35 °C, overnight), before being cooled back to room temperature.

In another oven-dried two-necked round-bottomed flask (100 ml), equipped with a stirring bar and connected to an argon filled Schlenk line, phosphorus trichloride (0.51 g, 12.35 mmol), lithium bromide (0.03 g, 1.24 mmol) and copper(I) iodide (0.07 g, 1.24 mmol) were added to degassed, dry diethyl ether (15 ml). The flask was placed in an acetone-dry ice bath (-78 °C), and the mixture was allowed to cool, with vigorous stirring.

The solution containing the Grignard reagent was transferred *via* a cannula filter into the PCl_3 solution, and stirred at -78 °C. The dry ice-acetone bath was then removed, and the reaction mixture was

brought to ambient temperature and left to react for a further 2 h with vigorous stirring on reaching this temperature. The solvent was removed under reduced pressure (25 °C, 10^{-2} bar) and the product was dissolved in pentane (25 ml). Degassed water (25 ml) was subsequently added, and the flask was vigorously shaken by hand, the organic layer was removed *via* cannula transfer into an oven dried flask (100 ml) and again washed with degassed water (25 ml). This was transferred *via* cannula into an oven dried flask (100 ml). Finally, the organic phase was dried using sodium sulfate and the liquid phase was transferred *via* cannula filtration into an oven dried flask (100 ml). The solvent was removed under reduced pressure in an ice bath (0 °C, 10^{-2} bar), to give a colourless liquid. ^{31}P NMR showed that multiple phosphorus environments were present (Figure S19).

Attempted synthesis of D_{68}-trihexyltetradecylphosphonium chloride

D_{39}-P_{666} (1.0 eq.) and D_{29}-1-tetradecylchloride (1.3 eq.) were added to a flask in acetonitrile and heated to reflux (60 °C) under argon for 1 week. ^{31}P NMR showed that multiple phosphorus environments were present.

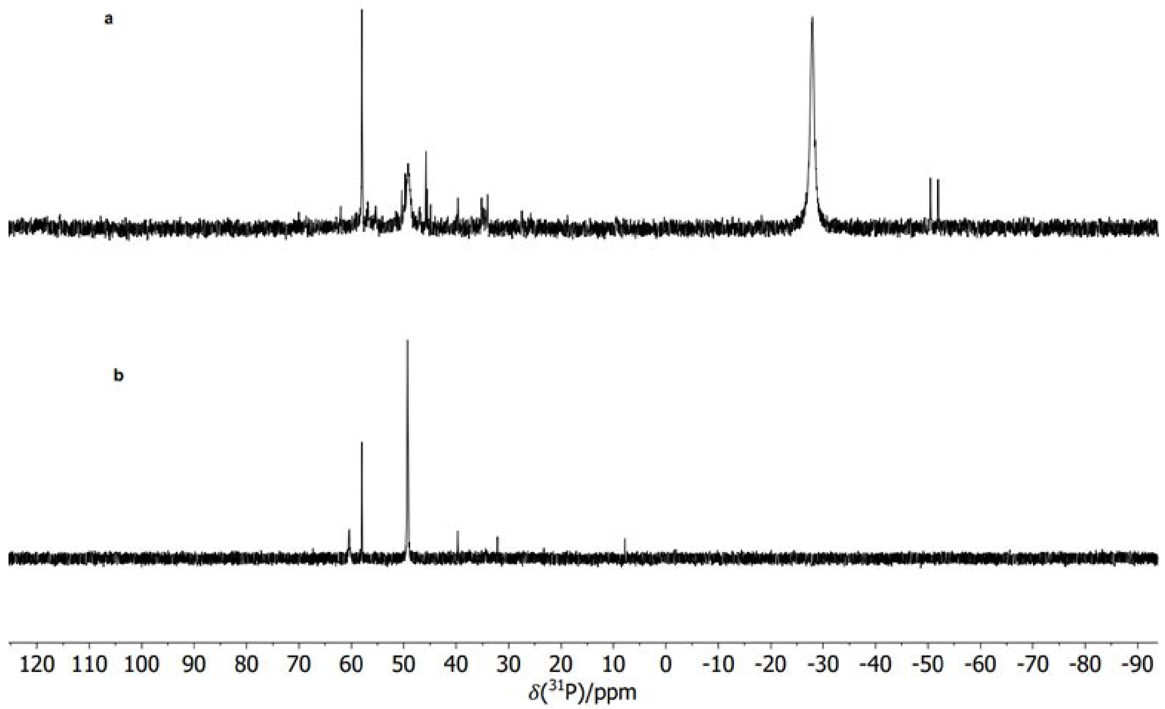

Figure S19. ^{31}P NMR spectra of a) attempted synthesis of D_{39}-P_{666} showing the presence of multiple species and b) alkylation with D_{29}-1-tetradecylchloride after one week.

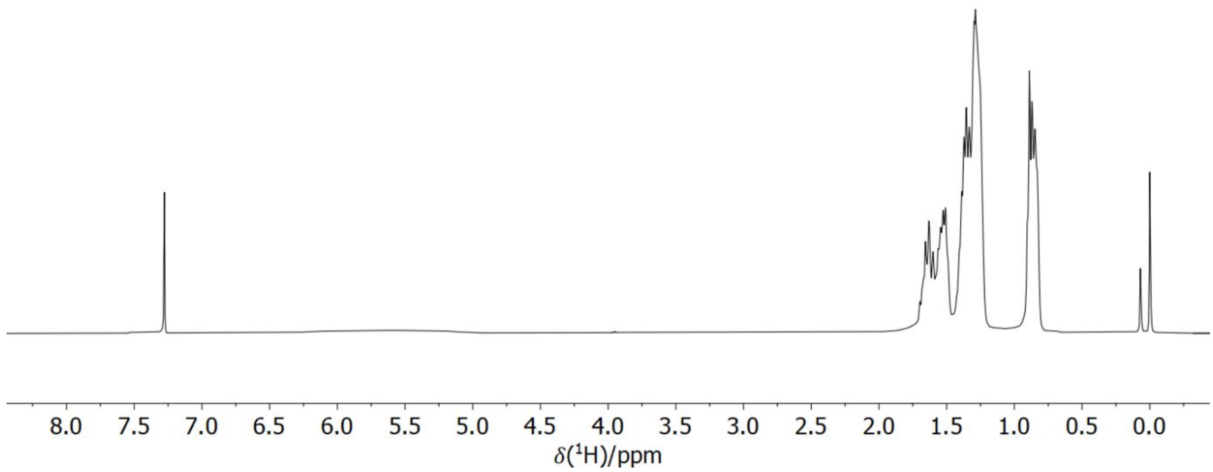

Figure S20. ^{1}H NMR of D_{39}-trihexylphosphine oxide in $CDCl_3$

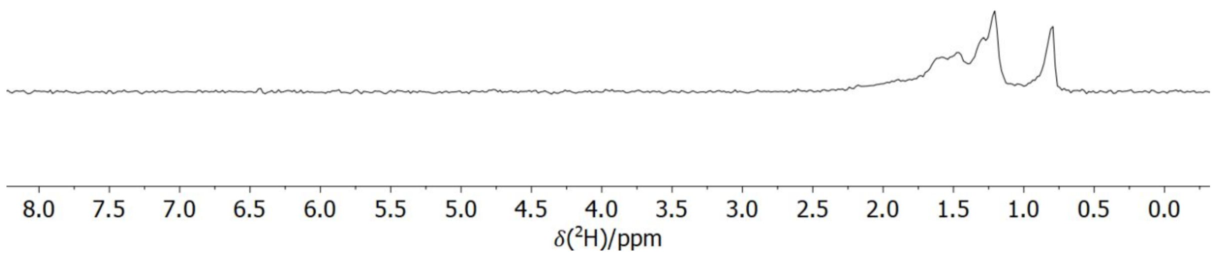

Figure S21. ^{2}H NMR of D_{39}-trihexylphosphine oxide

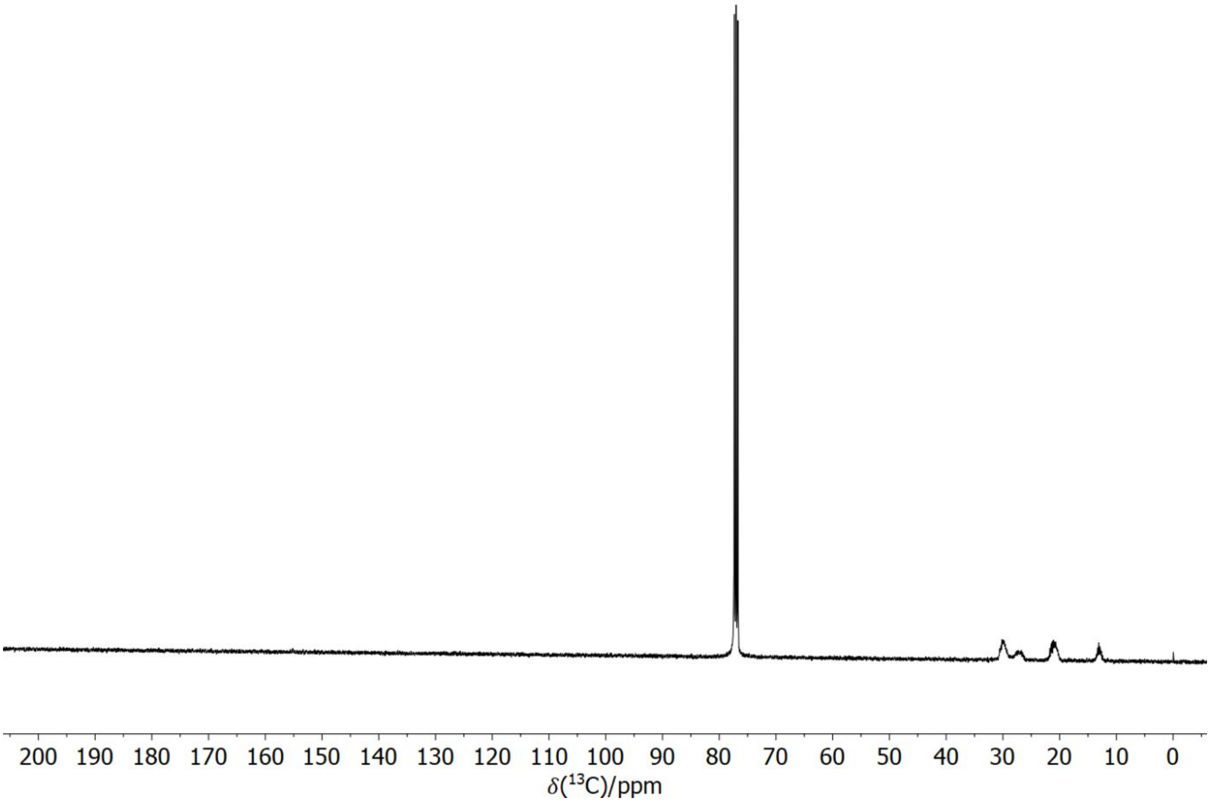

Figure S22. ^{13}C NMR of D_{39}-trihexylphosphine oxide in $CDCl_3$

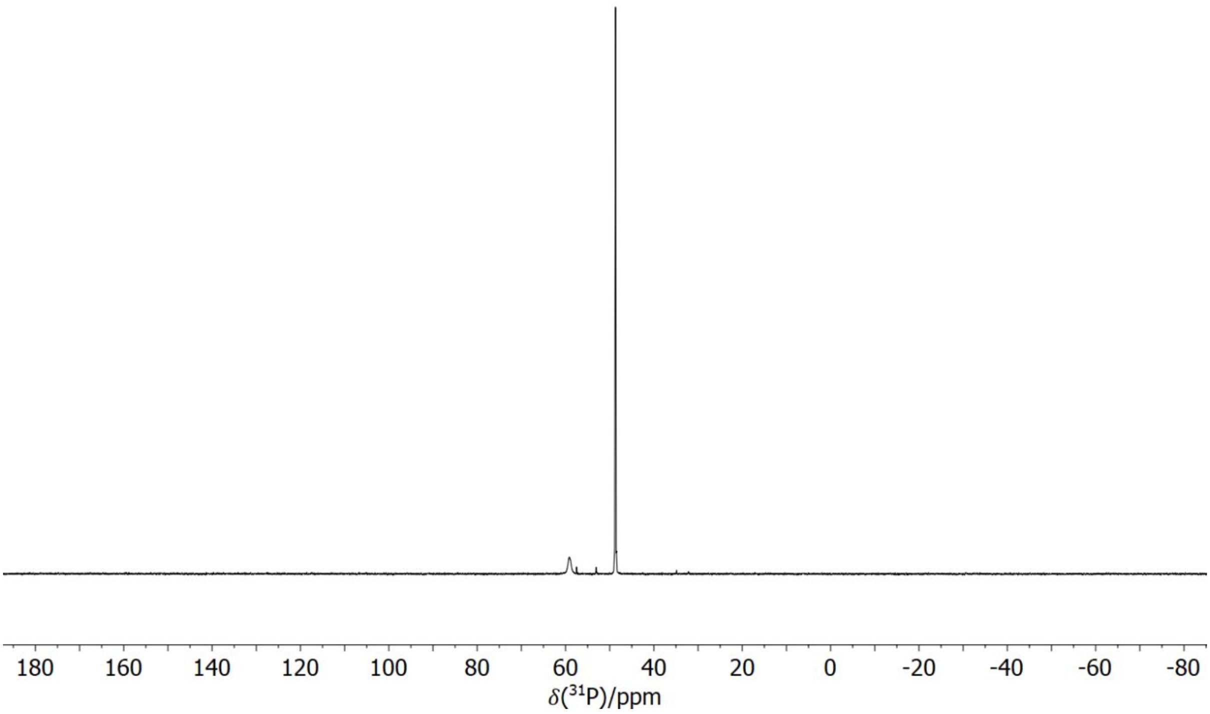

Figure S23. ^{31}P NMR of D_{39}-trihexylphosphine oxide in $CDCl_3$

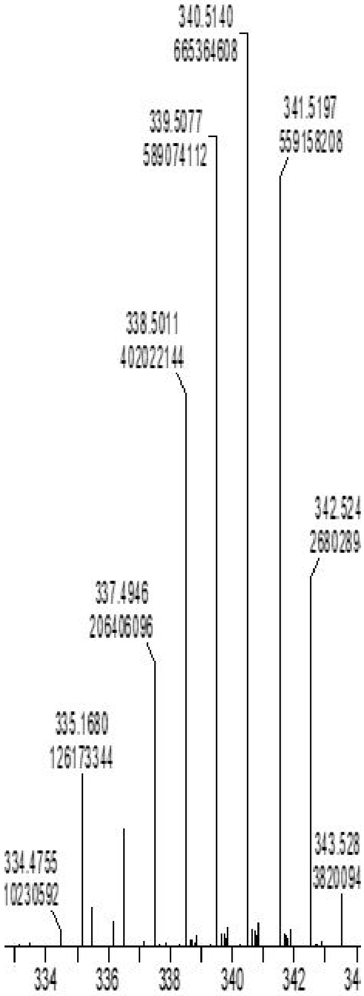

Figure S24. ESI-MS of D_{39}-trihexylphosphine oxide

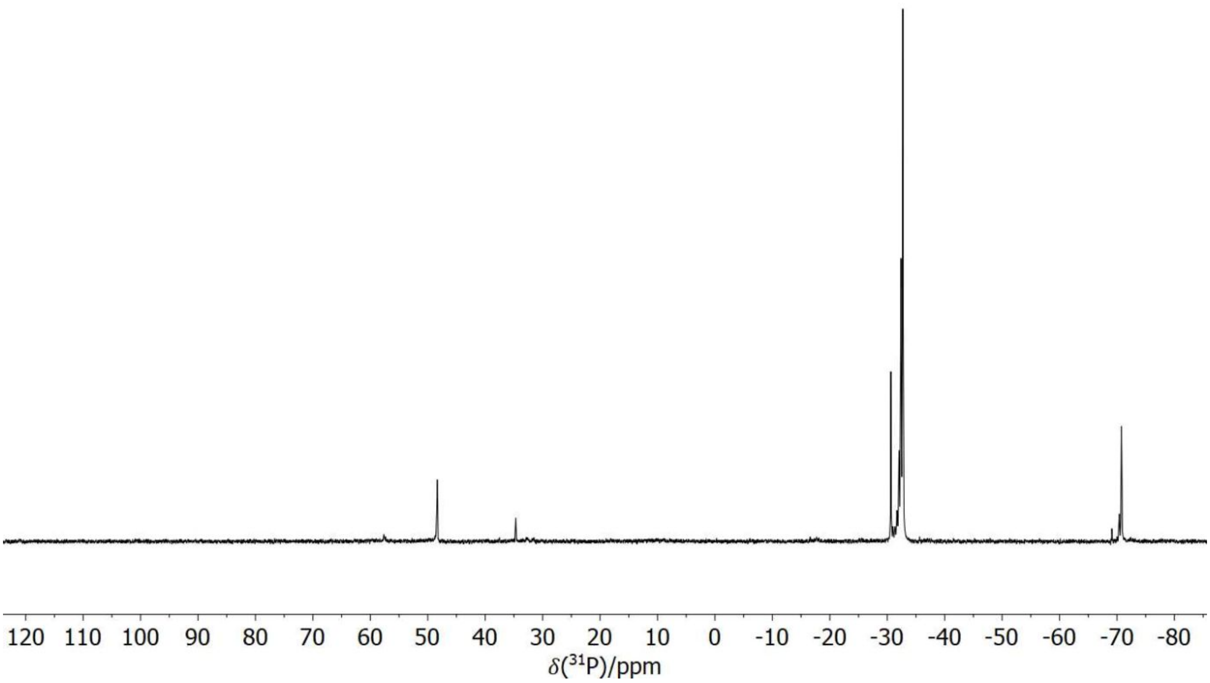

Figure S25. ^{31}P NMR of D_{39}-trihexylphosphine in $CDCl_3$ showing the product peak at -32.72 ppm and an impurity peak at -70.6 ppm.

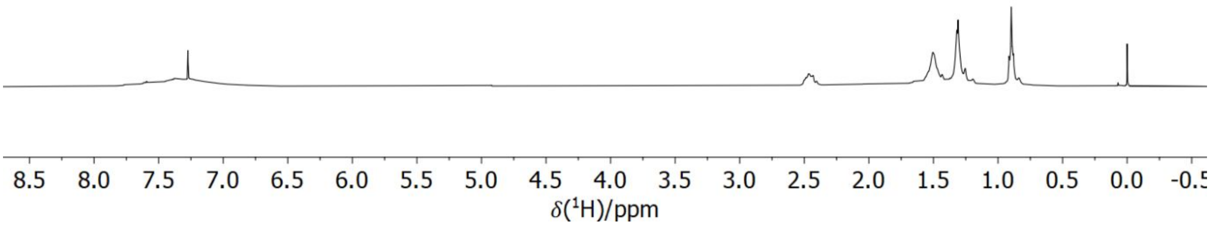

Figure S26. ^{1}H NMR of D_{68}-[$P_{666,14}$]Cl in $CDCl_3$

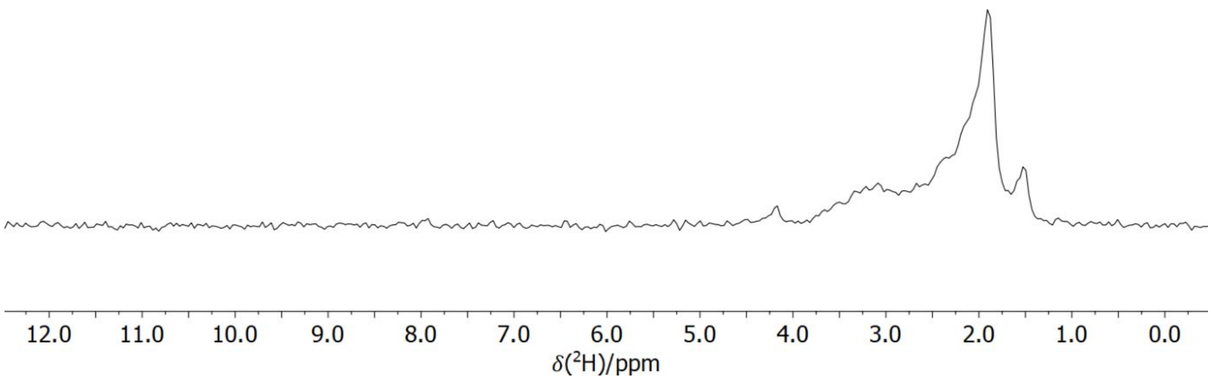

Figure S27. ^{2}H NMR of D_{68}-[$P_{666,14}$]Cl

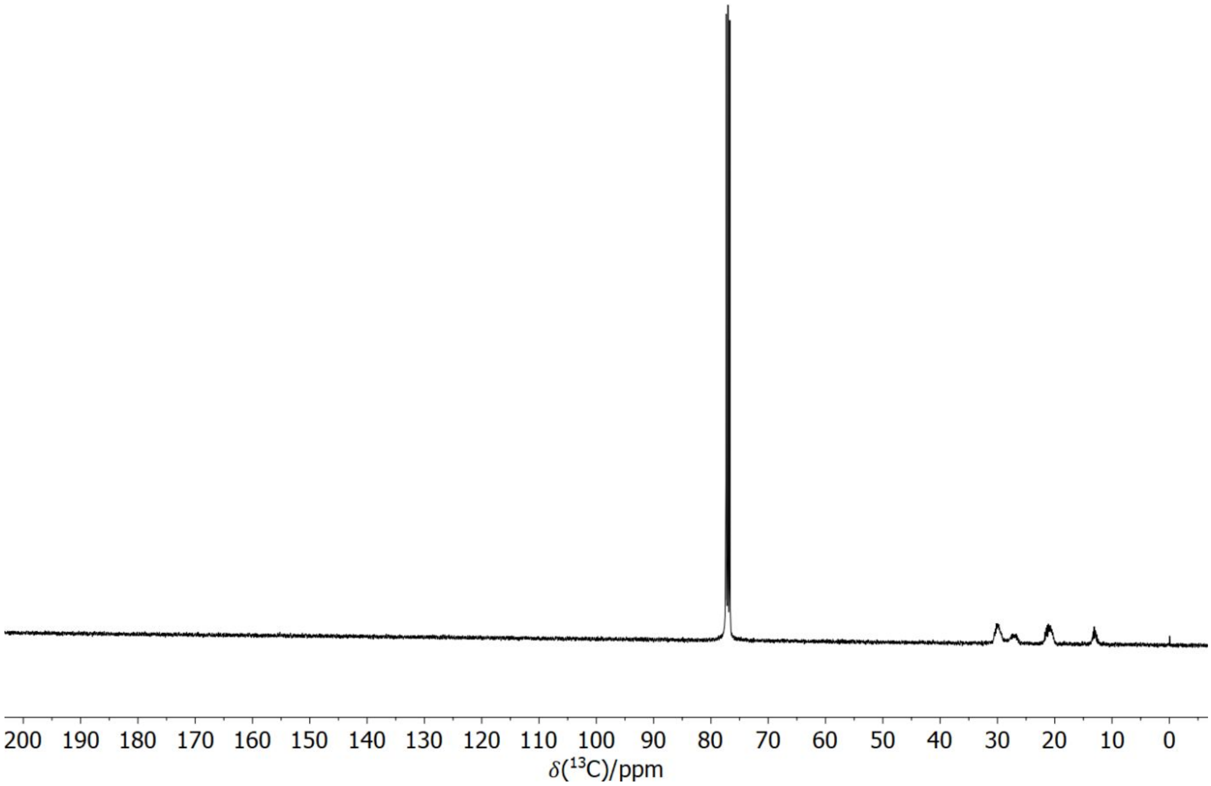

Figure S28. ^{13}C NMR of D_{68}-[$P_{666,14}$]Cl in $CDCl_3$

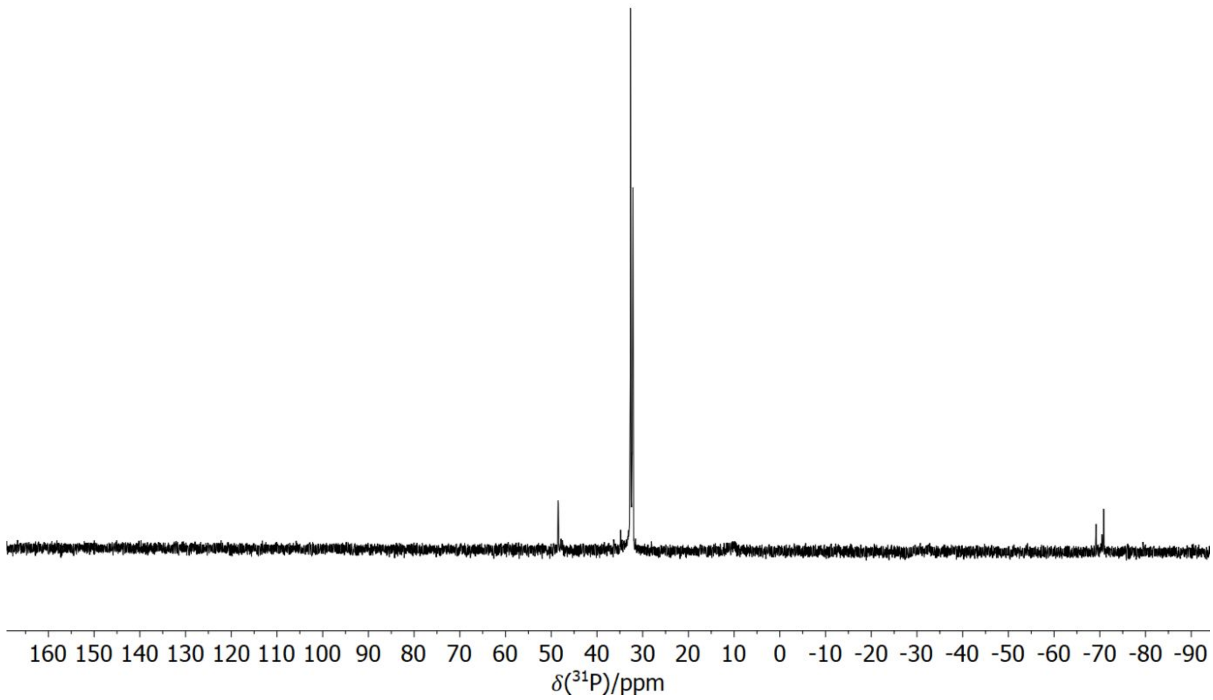

Figure S29. ^{31}P NMR of D_{68}-[$P_{666,14}$]Cl in $CDCl_3$

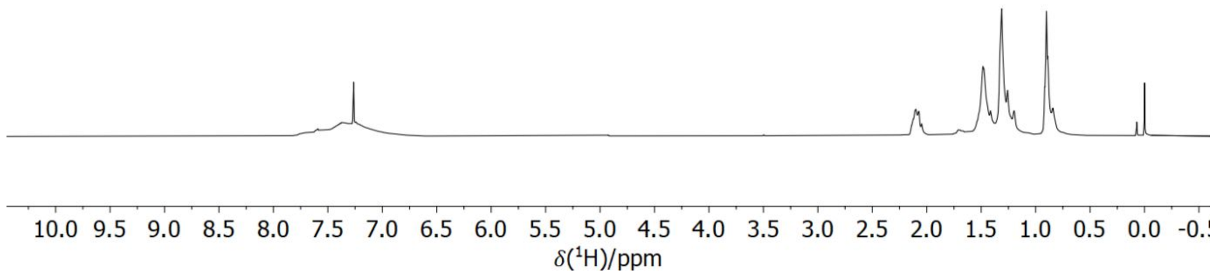

Figure S30. ^{1}H NMR of D_{68}-[$P_{666,14}$][NTf_2] in $CDCl_3$

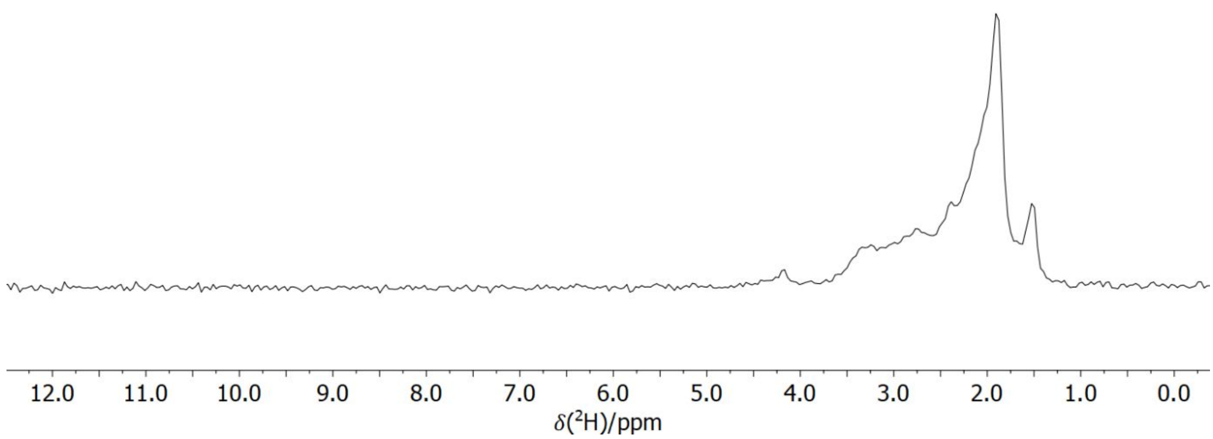

Figure S31. ^{2}H NMR of D_{68}-[$P_{666,14}$][NTf_2]

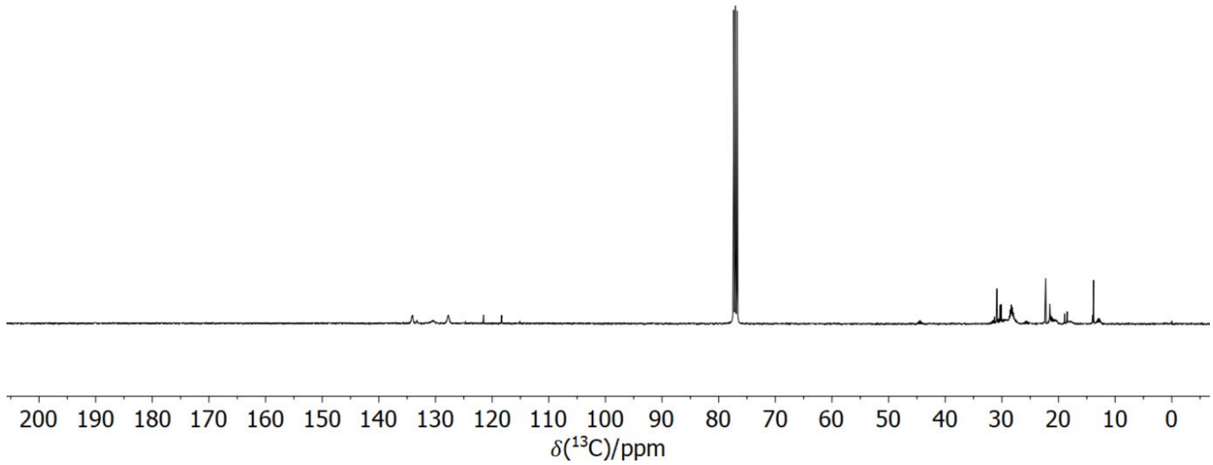

Figure S32. ^{13}C NMR of D_{68}-[$P_{666,14}$][NTf_2] in $CDCl_3$

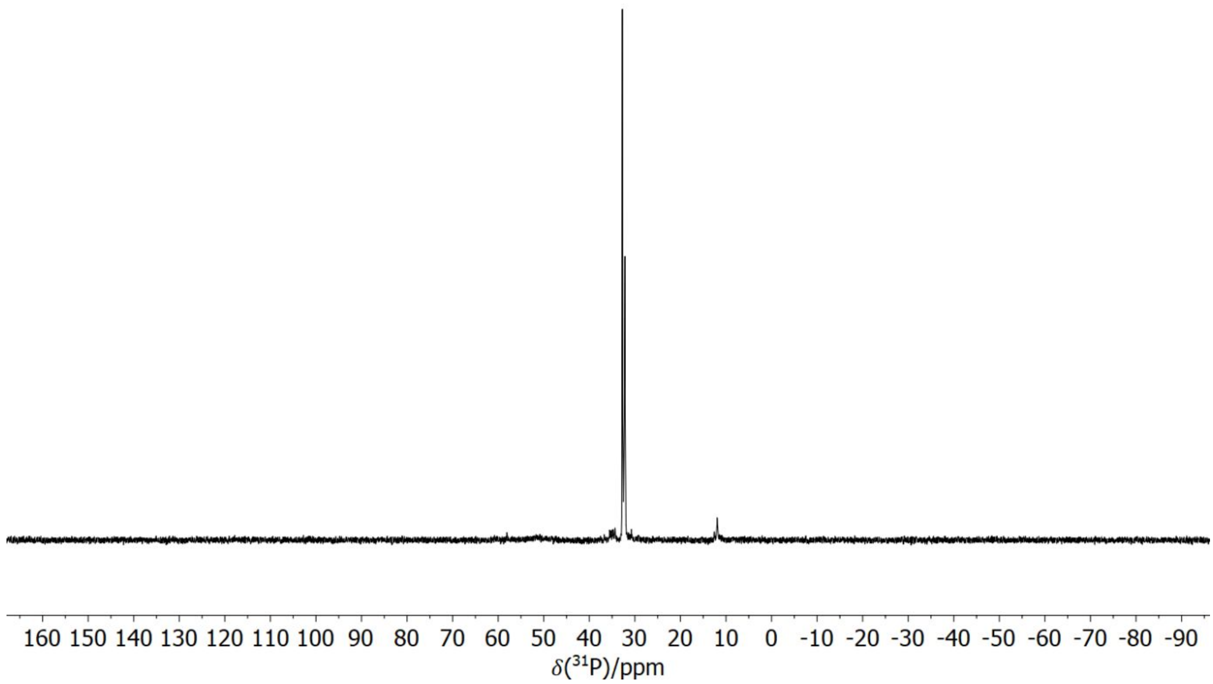

Figure S33. ^{31}P NMR of D_{68}-$[P_{666,14}][NTf_2]$ in $CDCl_3$

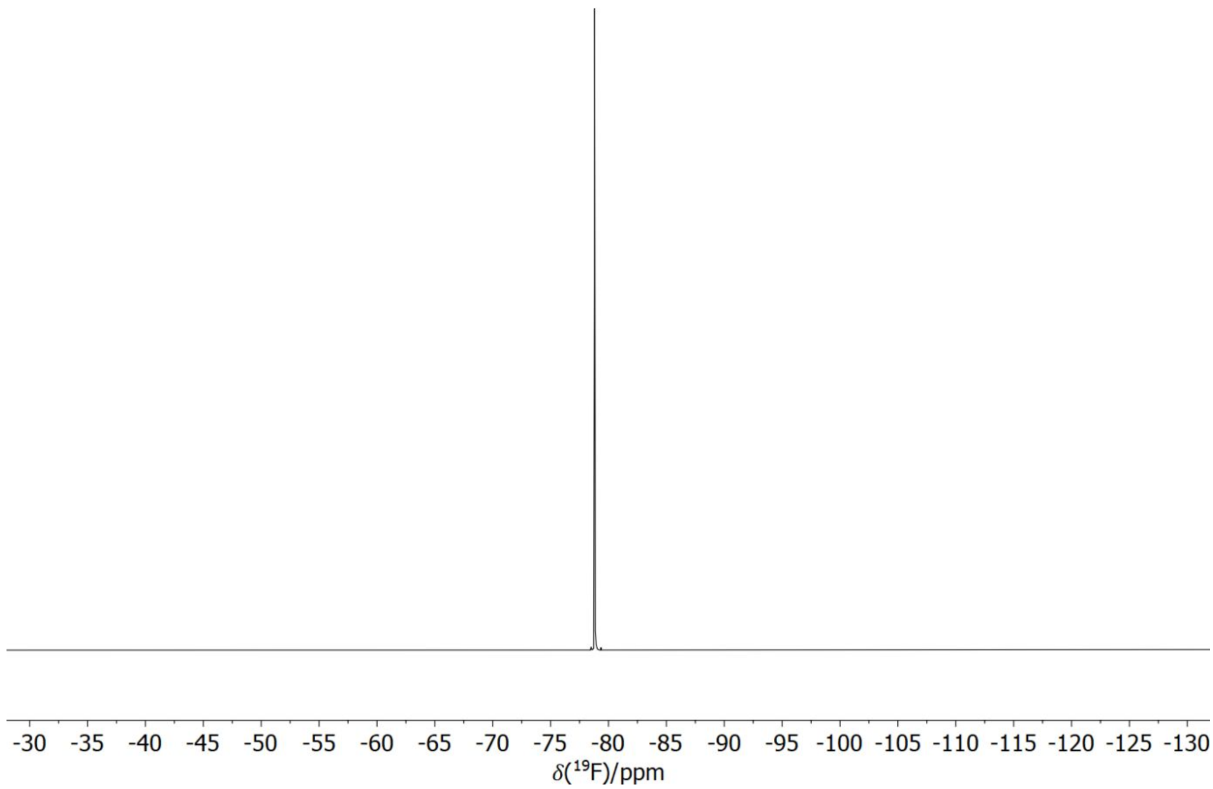

Figure S34. ^{19}F NMR of D_{68}-$[P_{666,14}][NTf_2]$ in $CDCl_3$

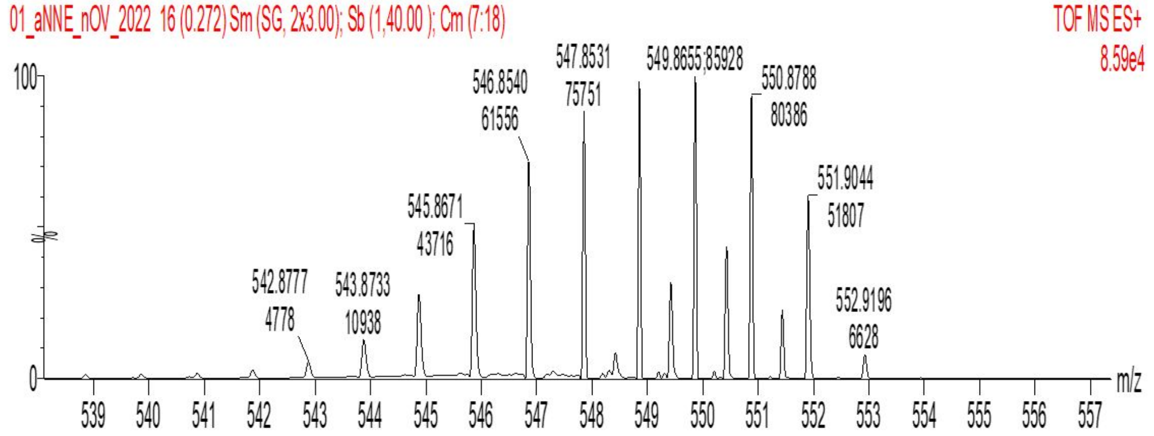

Figure S35. ESI-MS of D_{68}-[$P_{666,14}$][NTf_2]

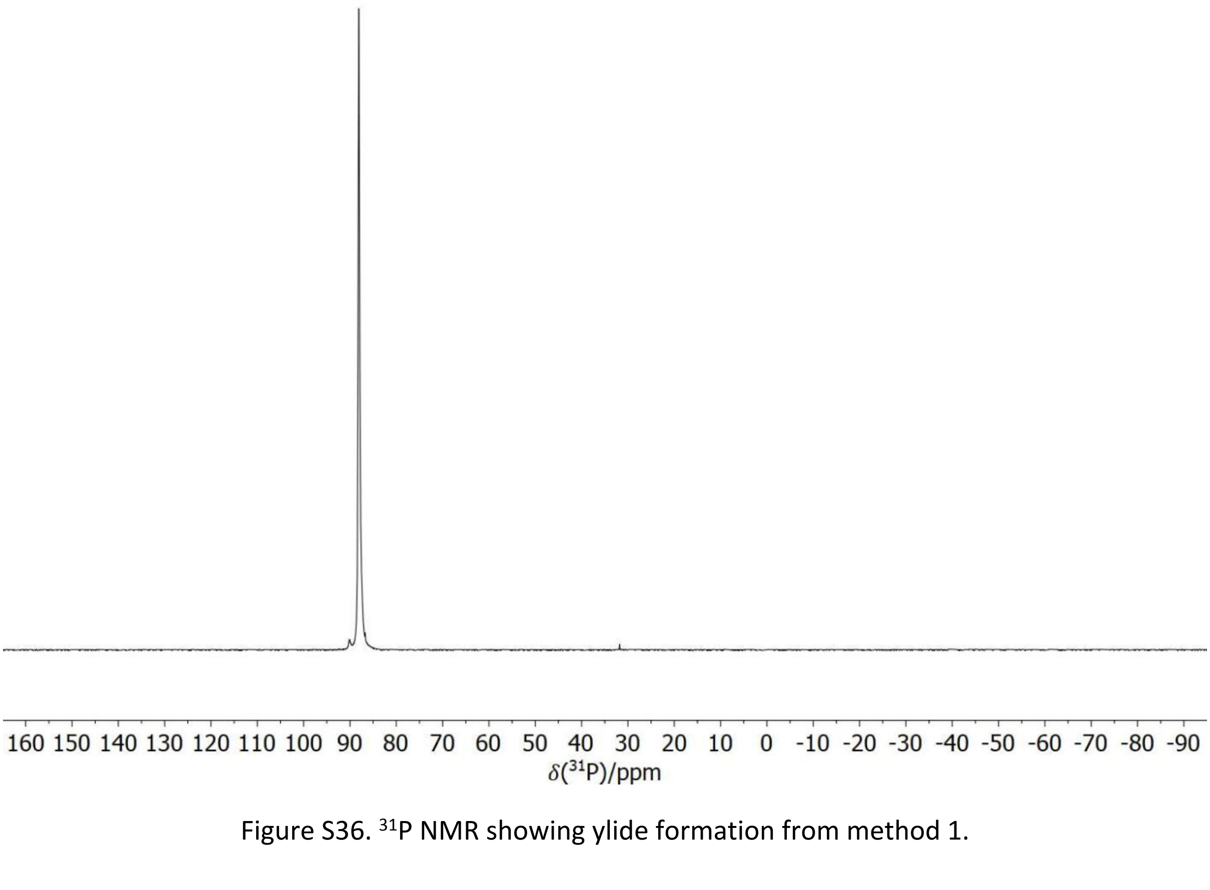

Figure S36. ^{31}P NMR showing ylide formation from method 1.

Neutron scattering experiments

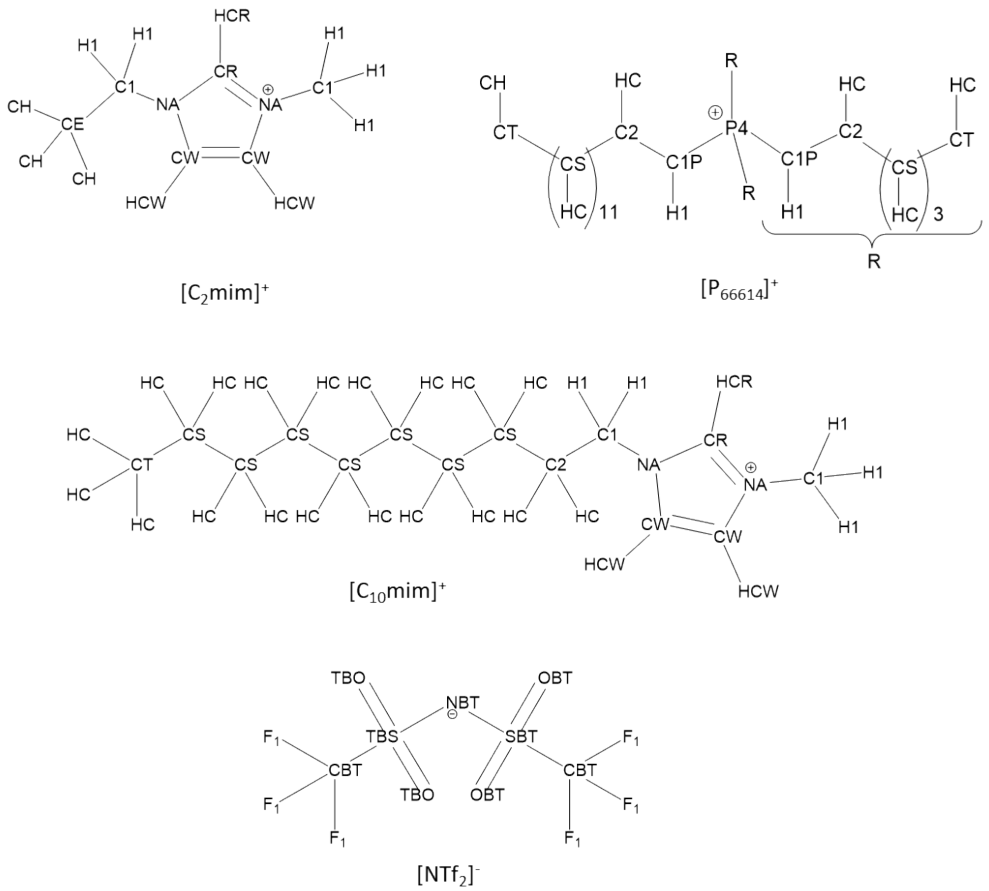

Figure S37. Molecular structures and atom types for $[C_2mim]^+$, $[C_{10}mim]^+$ and $[P_{666,14}]^+$ cation and $[NTf_2]^-$ anion.

Table S1. Simulation box size parameters.

Ionic liquid	Number of molecules (cation:anion)	Box size, n/ Å	Number density / atoms Å^{-3}
$[C_2mim][NTf_2]$	1000 (500:500)	59.66	0.080
$[C_{10}mim][NTf_2]$	1000 (500:500)	68.86	0.089
$[P_{666,14}][NTf_2]$	500 (250:250)	66.68	0.098

Table S2. Interatomic distances (taken from the first peak maximum in the site-site radial distribution functions) and relative coordination numbers (CN, calculated to the minimum after the first peak in the radial distribution functions) between different atom types for [C_2mim][NTf_2] and [C_{10}mim][NTf_2]. H_{CW1} refers to H_{CW} beside the alkyl chain and H_{CW2} refers to H_{CW} beside the methyl group.

	**[C_2mim][NTf_2]**		**[C_{10}mim][NTf_2]**	
**Interaction**	**Peak (minima)**	**CN**	**Peak (minima)**	**CN**
$H_{CR}\cdots O_{BT}$	2.7 (4.0)	0.65	2.7 (4.0)	0.52
$H_{CW1}\cdots O_{BT}$	2.7 (4.0)	0.54	2.7 (4.0)	0.41
$H_{CW2}\cdots O_{BT}$	2.7 (4.0)	0.58	2.7 (4.0)	0.46
$H_{1(met)}\cdots O_{BT}$	2.7 (4.0)	0.53	2.8 (4.0)	0.40
$H_{1(C2\ chain)}\cdots O_{BT}$	2.7 (4.0)	0.44		
$H_{C}\cdots O_{BT}$	2.8 (4.0)	0.33		
$H_{CR}\cdots N_{BT}$	3.0		3.1	
$H_{CW1}\cdots N_{BT}$	3.0		3.1	
$H_{CW2}\cdots N_{BT}$	3.0		3.1	
$O_{BT}\cdots H_{CR}$	2.7 (4.0)	0.63	2.7 (4.0)	0.50
$O_{BT}\cdots H_{CW1}$	2.7 (4.0)	0.55	2.7 (4.0)	0.41
$O_{BT}\cdots H_{CW2}$	2.7 (4.0)	0.59	2.7 (4.0)	0.46
$N_{BT}\cdots H_{CR}$	3.01		3.1	
$N_{BT}\cdots H_{CW1}$	3.01		3.1	
$N_{BT}\cdots H_{CW2}$	3.01		3.1	

Table S3. Interatomic distances (taken from the first peak maximum in the site-site radial distribution functions) and relative coordination numbers (CN, calculated to the minimum after the first peak in the radial distribution functions) between different atom types for [$P_{666,14}$][NTf_2].

Interaction	Peak	CN
$H_1 \cdots O_{BT}$	2.7 (3.7)	0.20
$H_1 \cdots N_{BT}$	3.1	
$O_{BT} \cdots H_1$	2.7 (3.7)	0.20
$N_{BT} \cdots H_1$	3.1	

Table S4. Three sets of charges used for the Dissolve model for [C_2mim][NTf_2].

Atom type	Charge CLP	Charge ESP	Charge LPG
CW	-0.094	-0.191	-0.058
NA	0.108	0.159	-0.195
CR	-0.080	-0.123	0.106
C1	-0.123	0.050	-0.019
CE	-0.036	-0.090	-0.236
H1	0.094	0.106	0.131
HCW	0.152	0.231	0.227
HCR	0.152	0.237	0.241
H1	0.094	0.014	0.128
HC	0.043	0.044	0.111

Table S5. Three sets of charges used for the Dissolve model for [C_{10}mim][NTf_2].

Atom type	Charge CLP	Charge ESP	Charge LPG
CW	-0.094	-0.199	-0.059
NA	0.108	0.180	-0.192
CR	-0.080	-0.155	0.104
C1	-0.123	0.036	-0.022
C2	0.007	0.136	-0.175
CS	-0.087	0.096	-0.151
CT	-0.130	-0.137	-0.207
HCR	0.152	0.244	0.241
HCW	0.152	0.232	0.227
H1	0.094	0.076	0.130
HC	0.043	-0.038	0.082

Table S6. Three sets of charges used for the Dissolve model for $[P_{666,14}][NTf_2]$.

Atom type	Charge CLP	Charge ESP	Charge LPG
P4	0.491	0.485	1.944
C1P	-0.224	-0.111	-0.624
CS	-0.087	0.089	-0.153
CT	-0.13	-0.107	-0.208
C2	0.007	0.171	-0.136
HC	0.043	-0.028	0.08
H1	0.094	0.045	0.138

EPSR modelling and fit to experimental data for the three charges sets

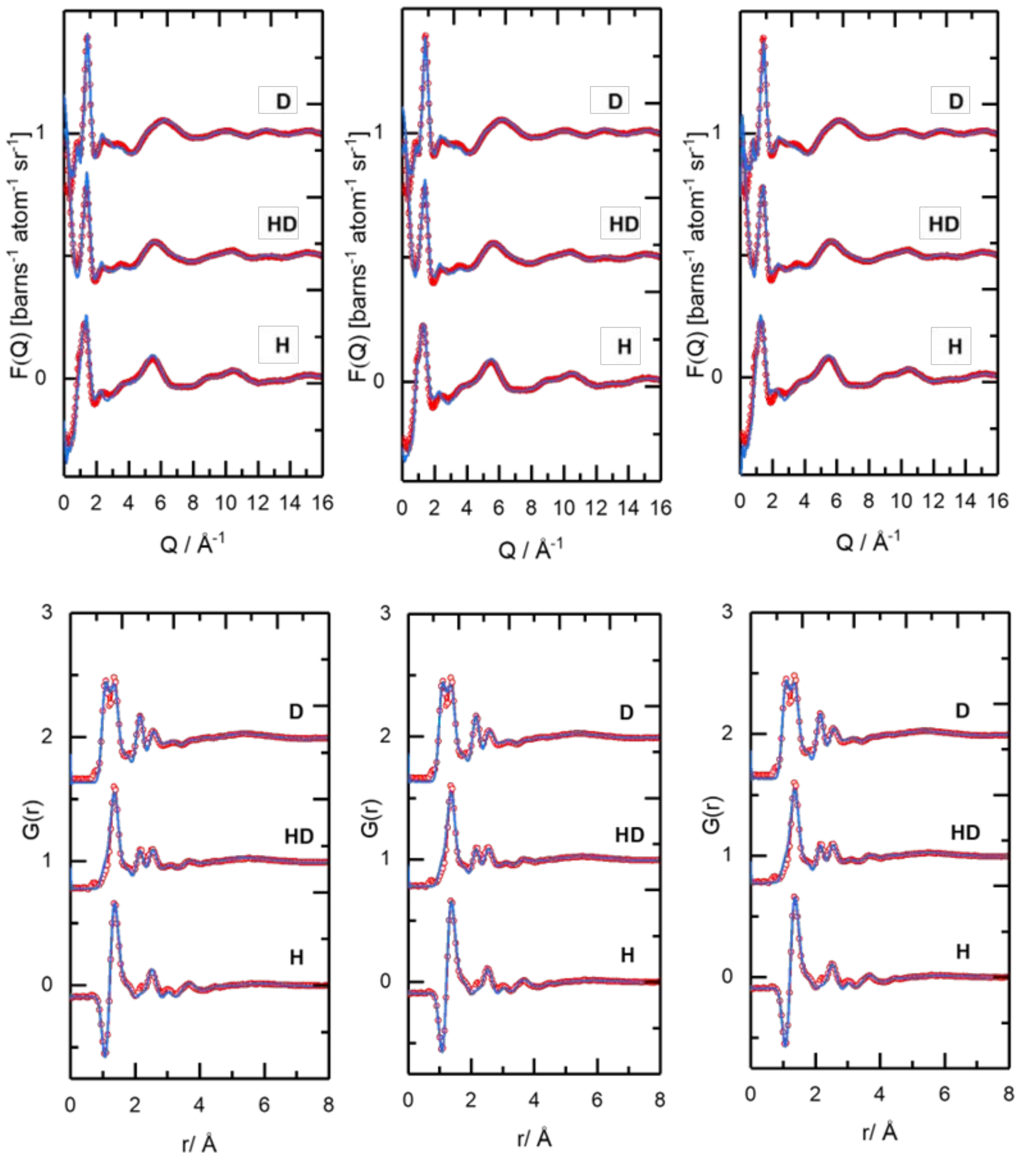

Figure S38. Total structure factors F(Q) (top), and the corresponding Fourier transform to real space G(r) radial distribution functions (bottom) showing experimental data (red symbols) and Dissolve modelled (blue solid line) for [C_2mim][NTf_2] for left: CLP charges, middle: ESP charges and right: LPG charges sets.

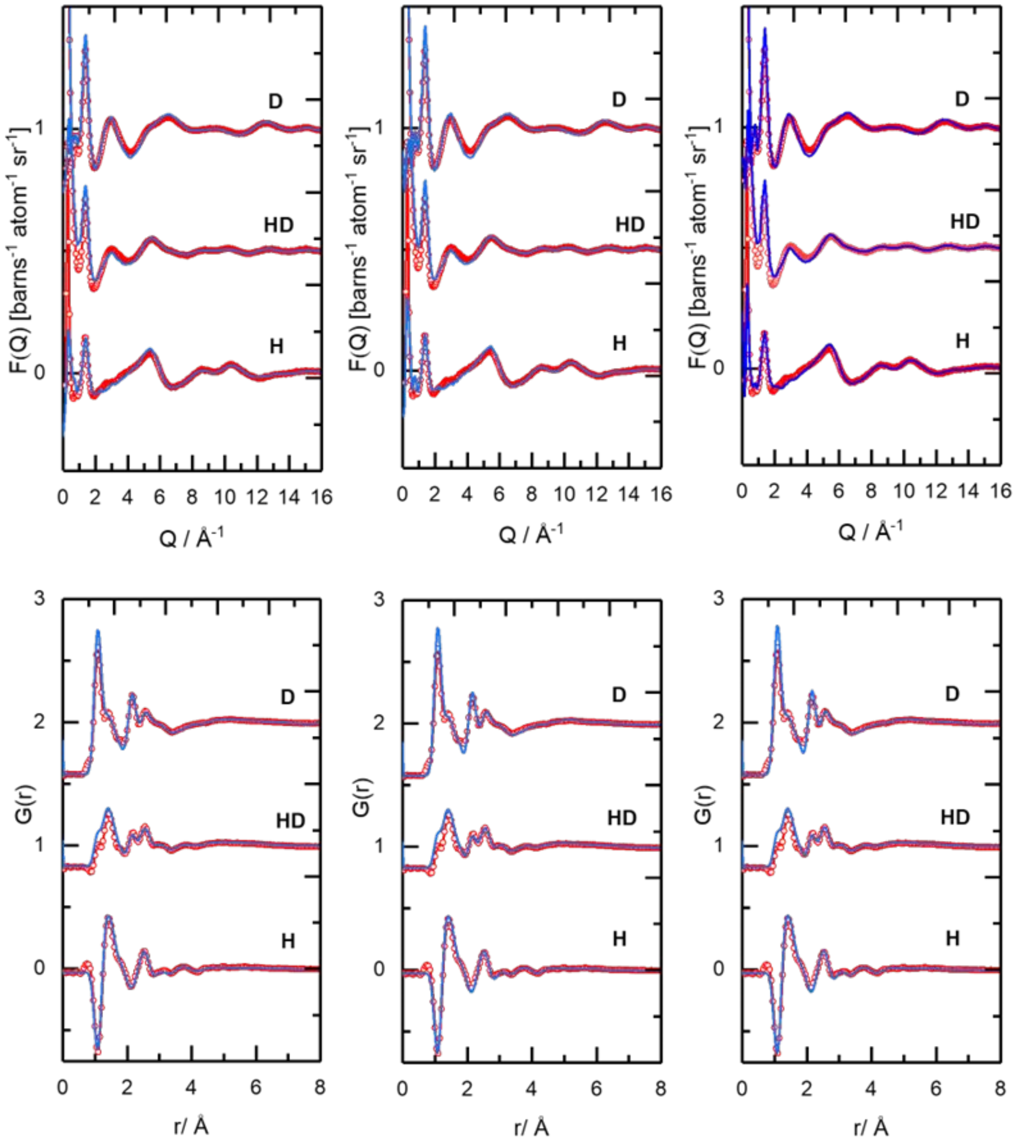

Figure S39. Total structure factors F(Q) (top), and the corresponding Fourier transform to real space G(r) radial distribution functions (bottom) showing experimental data (red symbols) and Dissolve modelled (blue solid line) for [C_{10}mim][NTf_2] for left: CLP charges, middle: ESP charges and right: LPG charges sets.

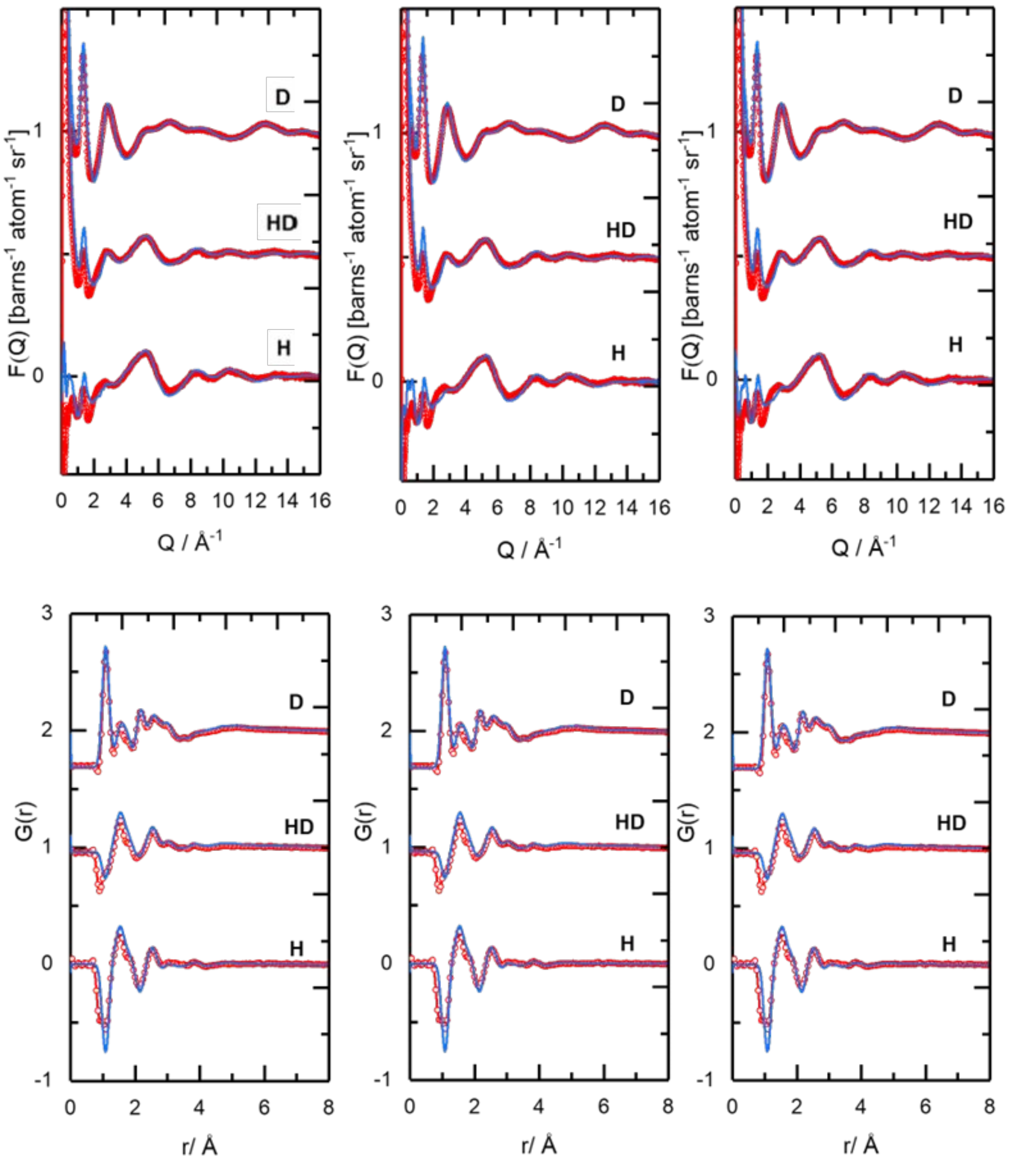

Figure S40. Total structure factors F(Q) (top), and the corresponding Fourier transform to real space G(r) radial distribution functions (bottom) showing experimental data (red symbols) and Dissolve modelled (blue solid line) for $[P_{666,14}][NTf_2]$ for left: CLP charges, middle: ESP charges and right: LPG charges sets.
